# Stimuli-responsive hydrogels for radiation-induced skin injury: from passive barriers to autonomous drug delivery systems

**DOI:** 10.1093/rb/rbag056

**Published:** 2026-03-13

**Authors:** Kai Zhang, Chulan Xiao, Yuanyuan Wang, Caitong Zhao, Zhenghang Dong, Zhihui Li, Xinmao Song, Chuanglong He, Yi Li

**Affiliations:** Department of Oncology, 920th Hospital of Joint Logistics Support Force, Kunming, Yunnan 650038, China; Department of Traditional Chinese Medicine, 920th Hospital of Joint Logistics Support Force, Kunming, Yunnan 650038, China; Department of Pathology, 920th Hospital of Joint Logistics Support Force, Kunming, Yunnan 650038, China; Department of Quality Control, the General Hospital of Northern Theater Command, Shenyang, Liaoning 110031, China; Department of Oncology, 920th Hospital of Joint Logistics Support Force, Kunming, Yunnan 650038, China; Department of Oncology, the General Hospital of Western Theater Command, Chengdu, Sichuan 610083, China; Department of Radiation Oncology, Ear, Nose & Throat Hospital of Fudan University, Shanghai 200031, China; College of Biological Science and Medical Engineering, Donghua University, Shanghai 201620, China; Yunnan Provincial Key Laboratory for Chest Disease Precision Medicine and Engineering, 920th Hospital of Joint Logistics Support Force, Kunming, Yunnan 650038, China; Kunming Medical University, Kunming, Yunnan 650500, China

**Keywords:** smart hydrogels, radiation-induced skin injury, stimuli-responsive biomaterials, theranostic platforms, AI-guided materials design

## Abstract

Radiation-induced skin injury (RISI) affects over 95% of radiotherapy patients. Current clinical management remains confined to passive supportive care, lacking mechanistic precision for RISI’s unique pathophysiology. This review adopts a function-centric perspective, classifying hydrogel systems across three generations: first-generation passive moisture barriers; second-generation bioactive platforms incorporating antioxidants, growth factors, stem cells and exosomes; and third-generation stimuli-responsive systems integrating autonomous drug release, self-healing capabilities and biosensor monitoring. We establish quantitative design thresholds by correlating RISI microenvironment parameters (pH 6.5–7.0, ROS 100–500 μM, MMP-9 elevation 5–10×) with responsive polymer specifications. Single-cell transcriptomic analysis has identified pro-inflammatory IL-17^+^ secretory fibroblasts and dysfunctional lymphatic endothelial cells as key dysregulated populations, thereby defining precise cellular targets amenable to hydrogel-based intervention. However, randomized trials demonstrate that certain hydrogel formulations unexpectedly prolonged healing, underscoring the need for design strategies based on quantitative pathophysiological insights rather than passive empiricism. We systematically examine enabling technologies—AI-guided materials optimization, 3D bioprinting and wearable biosensor integration—while addressing translational barriers including regulatory complexity, manufacturing scalability and standardized preclinical models. This framework provides actionable design principles to accelerate clinical deployment of next-generation hydrogels for millions of cancer survivors.

## Graphical Abstract

**Graphical Abstract:**
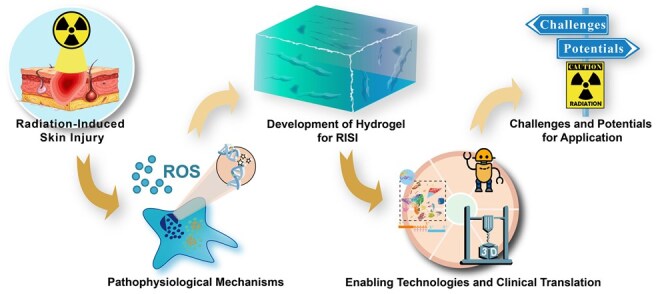

## Introduction

Radiation-induced skin injury (RISI) represents one of the most prevalent and debilitating complications of cancer radiotherapy, affecting over 95% of patients undergoing treatment [1, 2]. The pathophysiology of RISI extends beyond simple thermal burns, characterized by a unique cascade of persistent oxidative stress, chronic inflammation, progressive microvascular damage and pathological fibrosis—features that distinguish it fundamentally from conventional wounds [3, 4]. Current clinical management remains largely confined to passive wound care strategies, including topical corticosteroids, moisture-retentive dressings and symptomatic relief agents [5, 6]. However, these approaches fail to address the dynamic pathological microenvironment of RISI, which exhibits spatiotemporal heterogeneity in pH, reactive oxygen species (ROS) and matrix metalloproteinase-9 (MMP-9) [7, 8]. More critically, conventional topical formulations suffer from rapid clearance, insufficient tissue penetration and inability to synchronize drug release with disease progression, resulting in suboptimal therapeutic outcomes and, paradoxically, prolonged healing times in some cases. This therapeutic gap underscores the urgent need for precision drug delivery systems capable of autonomously adapting to the pathological microenvironment while providing sustained, localized therapeutic concentrations.

The evolution of drug delivery systems for RISI has progressed through three distinct paradigms, each representing a stepwise advancement in functional sophistication. First-generation approaches employed passive moisture-retentive hydrogels that primarily served as physical barriers, maintaining wound hydration but lacking active therapeutic components [9, 10]. While these systems reduced transepidermal water loss and provided mechanical protection, they failed to modulate the inflammatory and oxidative stress cascades central to RISI pathogenesis [8]. Second-generation platforms incorporated bioactive payloads—including antioxidants, anti-inflammatory agents and pro-regenerative factors—within hydrogel matrices [11]. For example, bio-inspired heparin-mimetic peptide hydrogels demonstrated significant antioxidant capacity for RISI repair [12] and multifunctional hydrogels loaded with nanoagents showed promise in improving the pathological microenvironment of radiation-combined wounds [7]. However, these systems relied on passive diffusion-mediated release kinetics, resulting in uncontrolled burst release, rapid depletion of therapeutic agents and frequent re-application requirements [13]. More problematically, the ‘one-size-fits-all’ release profiles could not accommodate the dynamic shifts in RISI microenvironment across healing stages (acute inflammatory phase vs. chronic remodeling phase) [14]. The emergence of third-generation stimuli-responsive hydrogels represents a paradigm shift toward precision-controlled drug delivery. These ‘smart’ systems integrate molecular sensors that detect pathological biomarkers and autonomously trigger drug release in a spatiotemporally synchronized manner [15]. By coupling therapeutic action to disease-specific cues, these platforms promise to overcome the limitations of passive release while minimizing systemic exposure and off-target effects.

Stimuli-responsive hydrogels leverage the pathological microenvironment of RISI as an intrinsic ‘trigger’ for on-demand drug release, fundamentally transforming passive dressings into autonomous therapeutic devices. Despite the growing body of literature on hydrogel-based wound healing, several key aspects warrant systematic analysis to advance rational design and clinical translation of controlled-release systems for RISI [9–11, 16]. First, while pH-responsive and ROS-responsive systems have been demonstrated, quantitative design frameworks that connect polymer pKa values, ROS-cleavable bond selection and enzymatic degradation kinetics to measure RISI microenvironment parameters remain underdeveloped [17]. Second, emerging single-cell transcriptomic datasets are revealing cellular heterogeneity in radiation-injured tissue—including distinct fibroblast and endothelial cell subpopulations [18]—yet their integration with precision drug delivery strategies remains nascent. Third, clinical translation experiences have revealed unexpected outcomes where hydrogel interventions failed to improve or even prolonged healing [5, 19], highlighting the complexity of matching material properties (release kinetics, mechanical characteristics) to wound healing dynamics [20].

A recent comprehensive review by Xu *et al*. [21] systematically summarized RISI pathogenesis and multifunctional biomaterial is from a material-centric perspective (stem cells/exosomes, hydrogels, nanomaterials, scaffolds). Adopting a parallel function-centric perspective, our work classifies systems by drug release control sophistication and explicitly links each generation to stage-specific clinical requirements of RISI healing. Together, these complementary frameworks can synergistically guide future therapeutic development from both material selection and functional design dimensions. Although alternative platforms such as liposome-based nanocarriers, metalloenzyme nanozymes and electrostimulation devices (e-skin) offer complementary advantages in subcellular targeting, catalytic antioxidant activity and real-time biophysical modulation, respectively, hydrogel systems uniquely integrate sustained localized drug residence, tuneable mechanical properties matching native tissue and capacity for multimodal cargo co-delivery (cells, proteins, nucleic acids and nanoparticles) within a single biocompatible matrix, making them particularly suited for managing the spatiotemporally complex pathophysiology of RISI across acute to chronic phases.

Based on a systematic search of PubMed, Web of Science and Scopus databases (2020–25) using keywords including ‘radiation-induced skin injury’, ‘hydrogel’, ‘stimuli-responsive’ and related terms, with AI-assisted tools (Claude Sonnet 4.5, Deepseek v3.2) employed to facilitate initial literature screening and categorization under author supervision, this review prioritizes peer-reviewed original research and clinical studies and contributes to these evolving areas by introducing a functional-complexity-based three-generation framework that systematically links hydrogel evolution with stage-specific RISI therapeutic needs: Generation 1 (passive barriers), Generation 2 (bioactive cargo delivery) and Generation 3 (intelligent stimuli-responsive systems). We compile quantitative microenvironment parameters from published RISI studies to establish design benchmarks for responsive systems. Drawing on emerging single-cell omics literature, we discuss potential precision therapeutic targets and corresponding delivery strategies. We analyze clinical translation challenges through representative case studies, examining relationships between material design choices and therapeutic outcomes. Finally, we explore how convergent technologies—AI-guided materials optimization, 3D bioprinting for spatial control and biosensor integration for feedback regulation—might be incorporated into future theranostic platforms.

## Pathophysiological mechanisms of RISI

**Figure 1:**
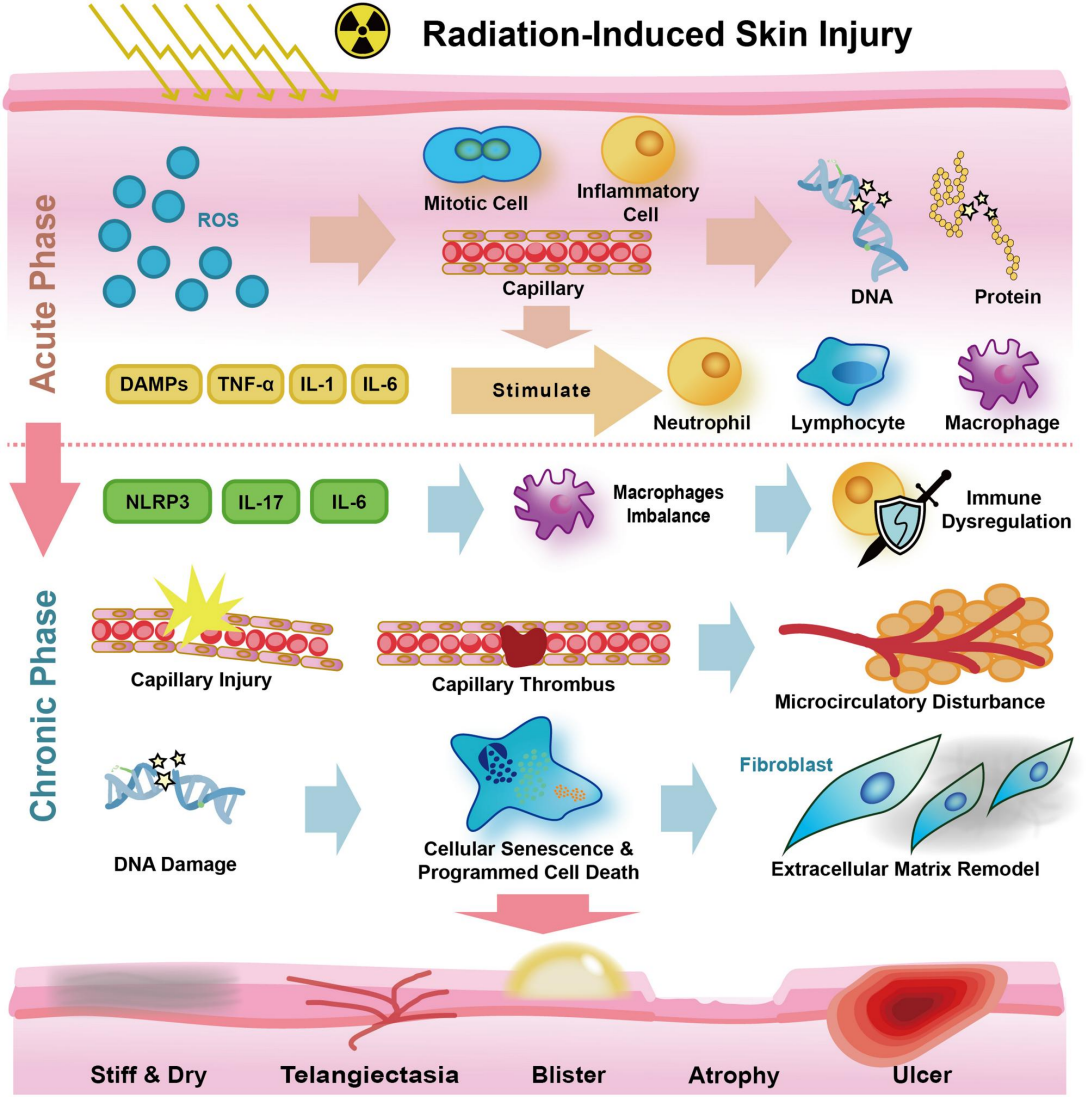
Brief pathophysiological mechanisms of RISI. ROS, reactive oxygen species; DAMPs, damage-associated molecular patterns; TNF, tumor necrosis factor; NLRP3, NOD-like receptor family pyrin domain containing 3.

Although RISI ultimately manifests as a hard-to-heal cutaneous lesion with persistent inflammation, oxidative stress, protease dysregulation, hypoxia and fibrotic remodeling, it should not be viewed as a generic ‘chronic wound’. Compared with metabolically driven ulcers such as diabetic foot ulcers (DFU) [22, 23] and other nonradiation skin injuries (e.g. pressure ulcers or thermal burns), RISI is uniquely initiated by ionizing radiation–induced DNA damage and mitochondrial dysfunction, accompanied by stage-dependent microenvironmental shifts (Table 1) and radiation-specific microvascular injury that predispose to progressive radiation-induced fibrosis (RIF) [24, 25]. These distinctions imply that RISI-targeted hydrogels must go beyond moisture management and conventional anti-inflammatory strategies, emphasizing stage-adaptive control of oxidative/inflammatory cascades, durable vasculoprotective/pro-angiogenic support under sustained hypoxia and early interception of senescence–fibrosis progression (Table 2 and Figure 1).

**Table 1:** Core pathophysiological distinctions between RISI and other skin injuries and the resulting hydrogel design implications.

Dimension	RISI characteristics	DFU and other injuries	RISI-specific hydrogel implications	Ref.
Initiating insult	Radiation-initiated injury with early genotoxic and mitochondrial stress	DFU is primarily metabolic/neuropathic/vascular and frequently infection-modulated	Radiation-tailored cytoprotection; mitochondria/DDR-linked protection	[23, 26]
Stage evolution	Strong spatial heterogeneity and radiation dose-dependent	Many chronic wounds show persistence	Prefer stage-adaptive and programmable release rather than a single fixed release profile	[14, 21]
Vasculopathy & Hypoxia	Direct endothelial injury and durable microcirculatory impairment are central drivers of chronicity	DFU ischemia is often systemic vasculopathy–embedded; pressure/burn vascular changes are injury-type specific	Emphasize durable vasculoprotection, pro-angiogenesis and hypoxia-aware strategies	[24, 27]
Immune programs	Persistent dysregulation with pathway-level nodes including inflammasome- and senescence-linked inflammatory loops	DFU inflammation often shaped by metabolic milieu, infection and biofilm	Prefer precision pathway-informed local immunomodulation rather than broad anti-inflammatory ‘blanket suppression’	[28, 29]
Late sequelae	High propensity for progressive radiation-induced fibrosis and stiff, poorly regenerative stroma	DFU is less defined by progressive RIF biology; burns scar via different pathways	Build in explicit anti-fibrotic/anti-senescence intent early, not only ‘close-the-wound’	[25, 30]

DFU, diabetic foot ulcer; DDR, DNA damage response; RIF, radiation-induced fibrosis.

**Table 2:** Quantitative microenvironment parameters of RISI and corresponding design benchmarks for stimuli-responsive hydrogels.

Pathological parameter	Normal tissue	Acute RISI (0–4 weeks)	Chronic RISI (>4 weeks)	Corresponding hydrogel design	Ref.
pH	7.4 (healthy skin)	6.5–7.0 (acidic inflammation)	7.15–8.9 (alkaline, chronic wounds)	pH-responsive polymers (pKa 6.8–7.2)	[31, 32]
ROS (H₂O₂)	<10 μM (baseline)	100–500 μM (peak at 3–5 days post-IR)	50–100 μM (persistent elevation)	Thioketal/diselenide ROS-cleavable bonds	[33, 34]
MMP-9	60.6 ng/mL (healthy)	259–862 ng/mL (acute inflammation)	552–630 ng/mL (chronic remodeling)	GPQGIAGQ peptide linkers	[35, 36]
Radiation dermatitis incidence	N/A	Grade 2: 49.8%, Grade 3: 2.2%	Moist desquamation: 21%	N/A	[37]
Oxygen saturation	6.19% (nonirradiated)	Hypoxia (<5% O₂)	Progressive decline	HIF-1α stabilizers, pro-angiogenic factors	[38]

These quantitative thresholds directly calibrate hydrogel responsiveness to measured RISI biomarkers, shifting from empirical formulation to precision material engineering.

### Acute phase: initial cascade reaction

#### Initial damage and oxidative stress

The most immediate effect after radiation exposure is the production of ROS and free radicals [39]. These highly active molecules can rapidly lead to oxidative stress, causing widespread damage to key components of skin cells such as DNA, proteins, lipids and carbohydrates. In particular, the rapidly dividing cells in the basal layer and capillaries are highly susceptible to radiation [26]. DNA damage, especially DNA double-strand breaks, is a key trigger for cell death or functional impairment [40]. If the repair mechanism is overwhelmed, DNA damage can lead to mutations, structural abnormalities and ultimately result in cell aging even death [41]. Therefore, hydrogel materials with ROS scavenging ability are a reasonable treatment approach.

#### Early inflammatory response

Damaged cells release large quantities of damage-associated molecular patterns (DAMPs) and pro-inflammatory cytokines—including tumor necrosis factor-α (TNF-α), interleukin-1 (IL-1), IL-6, chemokines, receptor tyrosine kinases and adhesion molecules [42]. These molecules attract immune cells (such as lymphocytes, macrophages and neutrophils) to infiltrate the damaged skin area. With the increased recruitment of neutrophils, the released cytotoxic granules further exacerbate the inflammatory state [43]. This persistent inflammation is a crucial driving factor for RISI progression to the chronic phase.

### Chronic phase: persistent injury

#### Chronic inflammation and immune dysregulation

If acute inflammation does not subside, it will transition to a chronic state [44]. This involves the sustained activation of inflammatory signaling pathways, such as NLRP3 inflammasome (involved in processing IL-1β and IL-18 and other pro-inflammatory cytokines) [45] and pathways involving IL-6 and IL-17[28]. The composition of immune cells will undergo changes, usually manifested as a persistent pro-inflammatory M1 macrophage phenotype or an imbalance between M1 and anti-inflammatory/promoting repair M2 macrophages, which hinders effective tissue repair [46]. The review by Xu *et al*. [21] provided a basic understanding of RISI pathophysiological mechanisms, describing it as a complex cascade reaction involving persistent injury signals, oxidative stress, chronic inflammation, immune dysregulation and microvascular injury. The gelatin methacryloyl (GelMA) particle hydrogel scaffold developed by Jaberi *et al*. [47] had a cell-level interconnected porous structure that could regulate macrophage polarization to a pro-healing phenotype and significantly improve wound healing quality in animal models.

#### Vascular injury and microcirculatory disturbance

Cutaneous endothelial cells are particularly sensitive to radiation [48]. Their damage leads to vascular inflammation, increased permeability and potential thrombosis. These vascular changes are the fundamental cause of edema and erythema observed in acute RISI [49]. With the accumulation of radiation dose and time, the vascular injury caused by ionizing radiation disrupts normal nutritional supply, exacerbates tissue hypoxia and promotes the fibrosis process. This microvascular injury is a sign of chronic RISI, which can lead to insufficient oxygen supply to skin cells, resulting in ulcers, wounds and skin necrosis [50].

#### DNA damage and epigenetic changes

Radiation can damage all cellular components and induce genetic and epigenetic changes, which play an essential role in the occurrence and progression of skin damage [51]. In the chronic phase, inflammation and oxidative stress can lead to various of changes in the cytokines, cell cycle and DNA damage, resulting in a cascade reaction of late responses and higher risk of skin cancer [52].

#### Cell death and cellular senescence

Radiation-induced cell damage leads to various forms of cell death, particularly the programmed cell death which depends on the mitogen-activated protein kinase (MAPK) system [53]. Furthermore, cellular senescence in epidermal keratinocytes is an important marker of radiation dermatitis [54]. As part of the epidermal stress response, these senescent keratinocytes will secrete pro-inflammatory mediators, thereby aggravating skin inflammation [55].

#### Fibrosis and ECM remodeling

The transition of RISI from acute phase to chronic phase not only results in not only the persistent inflammation, but also the ECM fibrotic response [56]. The inflammatory environment stimulates fibroblast activation and proliferation, leading to excessive production of collagen and other fibrous materials, which in turn triggers extracellular matrix remodeling [57]. The accumulation of ECM components in the dermis significantly increases tissue hardness and decreases skin elasticity, manifesting as skin stiffness and dryness. This phenomenon, known as RIF, is a severe, progressive and irreversible late-stage complication [25]. Histologically, chronic RISI is characterized by an increase in dermal cells (mainly fibroblast proliferation), thickening and disorganization of collagen bundles and disordered arrangement of ECM. These changes lead to thickening of the dermis, filled with dense fibrotic ECM replacing the normal skin structure [58].

## Development of hydrogel technology for RISI

**Figure 2:**
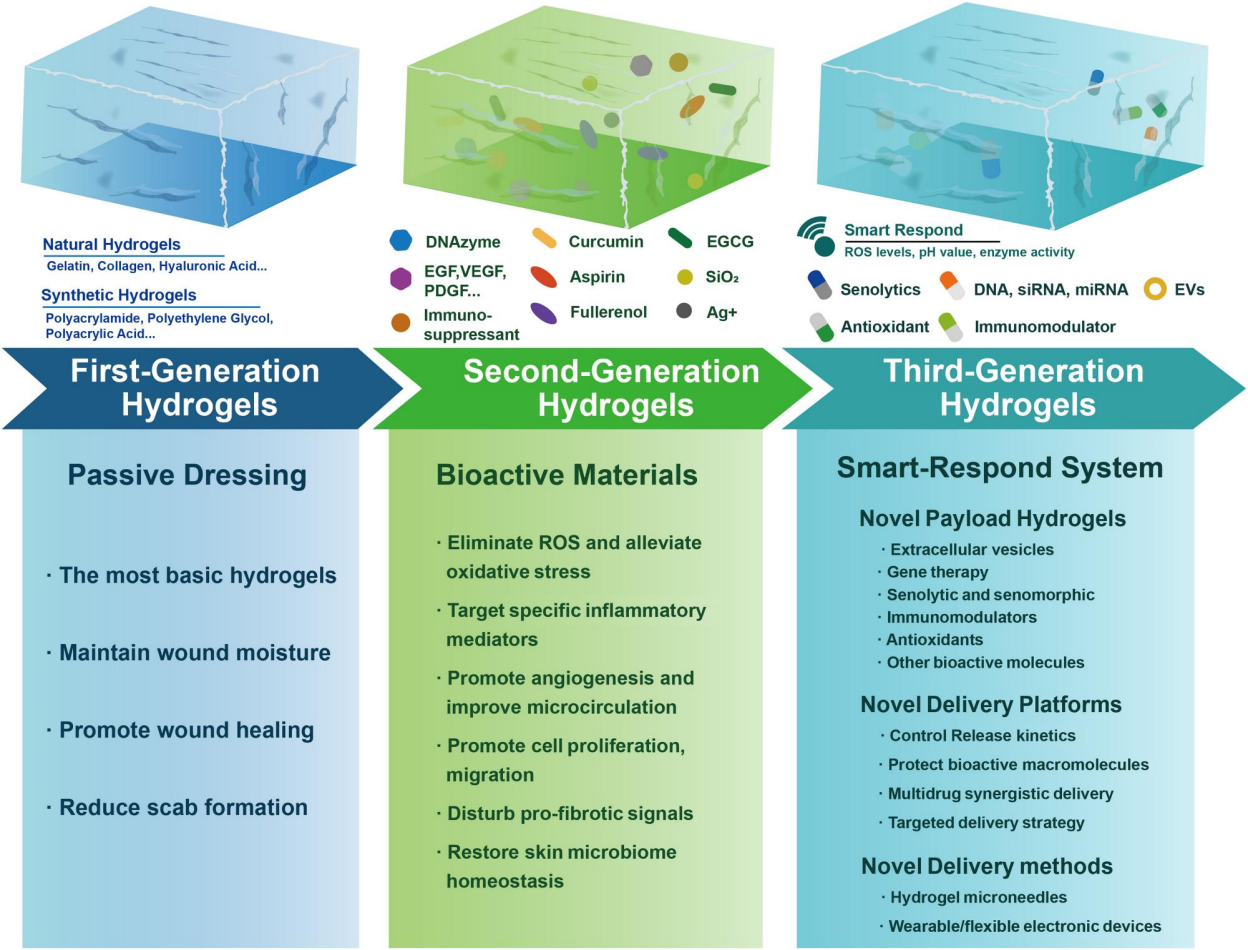
The technology development of hydrogel for RISI. The application of hydrogel in repair of RISI has undergone several generations of development, each bringing significant advancements in function and therapeutic effect. EVs, external vesicles; EGCG, epigallocatechin gallate.

The application of hydrogels in RISI management has undergone a paradigm shift from passive symptom management to active pathophysiology modulation and intelligent therapeutic intervention. This evolution reflects the deepening understanding of RISI pathobiology and advances in biomaterial science. Each generation needs progressively refined alignment between hydrogel design and RISI pathological demands—from passive moisture retention addressing barrier dysfunction (First-generation, 1G), to bioactive cargo delivery targeting oxidative stress and inflammation (Second-generation, 2G), to intelligent responsive systems synchronized with dynamic microenvironmental cues (Third-generation, 3G; Figure 2).

Hydrogels are three-dimensional polymer network materials made from natural or synthetic materials, characterized by high water content, good biocompatibility and high flexibility [10]. First-generation hydrogels developed primarily from the 1960s to early 2000s, functioned as passive physical barriers providing moisture retention, mechanical protection and pain relief through high water content (70–90%) and cooling effects. The 1G hydrogels we commonly recognize can be divided into three categories: natural polymer hydrogels, synthetic polymer hydrogels and composite hydrogels [59]. Natural hydrogels are based on natural materials such as gelatin [60], chitosan, sodium alginate [61], collagen, hyaluronic acid [62] and chondroitin sulfate, most of which are extracted from animals or marine organisms and these hydrogels generally have lower mechanical properties [63]. Synthetic hydrogels are primarily based on chemical raw materials such as polyacrylamide, polyethylene glycol, polyacrylic acid, polyvinyl alcohol, poly hydroxyethyl methacrylate and their derivatives. Although these hydrogels have controllable structures and excellent mechanical properties, they may produce biotoxicity during the crosslinking process [64]. Composite hydrogels are hydrogel materials which combine natural polymer materials with synthetic polymer materials, allowing for the integration of the advantages of both, thereby greatly expanding the application of hydrogels in the medical field. While these systems improved patient comfort compared to dry dressings, their clinical utility in moderate-to-severe RISI (Grade ≥ 2) proved limited. Clinical trials revealed that conventional first-generation hydrogels showed variable efficacy, with some formulations demonstrating benefit while others failed to improve outcomes compared to standard care [65], likely due to differences in wound exudate management capabilities and lack of bioactive modulation of chronic inflammation or vascular compromise.

Second-generation hydrogels emerging around 2000–15, transcended passive barriers by incorporating bioactive payloads to actively modulate RISI pathophysiology. These advanced systems deliver antioxidants such as EGCG and curcumin [66], anti-inflammatory agents including DNAzyme targeting NLRP3 inflammasome [21], pro-angiogenic factors like VEGF and deferoxamine [22], growth factors such as EGF and PDGF [23], stem cells and exosomes [67]. The therapeutic paradigm shifted from merely protecting the wound bed to reprogramming the healing process by targeting specific pathological axes: ROS scavenging breaks the oxidative stress cycle, inflammation suppression via NLRP3/IL-17/NF-κB pathway inhibition, angiogenesis promotion rescues microvascular injury and ECM remodeling prevents fibrosis [68]. However, second-generation systems face a critical limitation—static drug-release kinetics poorly matched to dynamic wound microenvironment changes. For instance, early-phase RISI (Days 0–7) requires rapid antioxidant release to combat acute oxidative stress, while chronic-phase injuries (beyond 4 weeks) benefit from sustained anti-fibrotic therapy [30]. Most second-generation hydrogels employ simple sustained release with first-order or zero-order kinetics that cannot adapt to these temporal demands.

Third-generation hydrogels, developed from approximately 2015 to present, represent intelligent therapeutic platforms that autonomously sense pathological cues and respond dynamically [69]. These smart systems detect microenvironmental changes including acidic pH (6.5–7.0 vs. physiological 7.4), elevated ROS (100–500 μM H_2_O_2_) and upregulated proteases such as matrix metalloproteinases MMP-2 and MMP-9 that are elevated 5- to 10-fold in chronic wounds [70], triggering on-demand drug release or property modulation. Recent innovations include X-ray responsive drug-free antioxidant hydrogels formed by copolymerization of disulfide-containing hyperbranched polymers that exhibit continuous antioxidant activity through oxidation of disulfide bonds and triggered release of growth factors after radiation-responsive network breakage [71]. Beyond stimuli-responsiveness, third-generation platforms integrate advanced functionalities such as self-healing hydrogels that restore mechanical integrity after damage [72], real-time monitoring through wearable biosensors for continuous wound status assessment [73] and 3D bioprinting enabling personalized tissue reconstruction with multilayered architecture [74]. The frontier of this generation involves closed-loop theranostic platforms combining biosensing, adaptive therapeutics and AI-guided decision-making to mimic tissue-level intelligence [73]. This evolutionary trajectory reflects increasing therapeutic precision: first-generation systems broadly support healing through nonspecific moisture provision, second-generation systems target specific pathways, while 3G systems mimic tissue intelligence through autonomous response to microenvironmental dynamics. Most clinically available products remain first-generation or simple second-generation formulations, while advanced third-generation systems are predominantly in preclinical research with translational barriers including manufacturing complexity, regulatory uncertainty for combination products and lack of standardized preclinical models [75].

### First-generation hydrogels: passive dressing

First-generation hydrogels primarily serve as passive dressings, addressing the fundamental need for barrier restoration and hydration maintenance in acute RISI, with their core function being to provide and maintain a moist healing environment for wounds [76]. This moist environment is crucial for promoting cell migration, proliferation and preventing wound drying, which is considered a significant advantage over traditional dry dressings.

The most basic hydrogels effectively maintain wound moisture and promote wound healing and reduce scab formation through their high-water content. They possess cooling properties, capable of covering exposed nerve endings, thereby alleviating pain and reducing discomfort during dressing changes. These hydrogel materials can soften and loosen necrotic tissue, aiding in autolytic debridement, making it easier to remove necrotic tissue during dressing changes. In addition, most of these hydrogels have good biocompatibility, minimizing adverse reactions and promoting the natural healing process [77]. Meanwhile, they are generally easy to apply to various wound shapes, including irregular or difficult-to-treat wounds.

However, for wounds with excessive exudate, these hydrogels may lead to peri-wound tissue maceration, requiring additional absorbent dressing coverage. Furthermore, some of these hydrogel dressings are nonadhesive and require secondary dressing or tape for fixation, which may cause further damage to the fragile skin affected by radiation. Therefore, many 1G hydrogels, especially those based on natural polymers, have poor mechanical strength, making them prone to rupture or deformation, which limits their application in wounds that require structural support [59]. Common examples include simple hydrogel dressings based on sodium alginate [61], hyaluronic acid [62] or gelatin [60]. For instance, the commercially available RadiaPlex hydrogel is based on aloe vera and hyaluronic acid, designed to just provide a moisturizing barrier and support natural healing [65]

Besides, 1G hydrogels lack bioactive control over the multi-axis pathology of RISI—namely oxidative stress, dysregulated immunity, microvascular compromise and fibroblast–myofibroblast remodeling, therefore, lacking the active therapeutic functions such as precision drug delivery, antibacterial or regeneration-promoting effects [78]. As a result, the clinical benefits of 1G hydrogels in high-risk cohorts are limited, especially under high surface dose and concurrent systemic therapy. This gap motivates the transition toward bioactive and synergistic designs in 2G and 3G platforms that proactively modulate various intertwined axes rather than merely shielding the wound.

### Second-generation hydrogels: active biomaterials

Second-generation hydrogels transcend simple passive dressings by integrating active biomaterials and therapeutic agents, responding to therapeutic needs for combating oxidative stress and inflammatory dysregulation documented in RISI pathogenesis, achieving more proactive wound management. Based on the pathophysiological mechanisms of RISI, second-generation hydrogel materials can identify and develop effective therapeutic targets. These hydrogels can be loaded with growth factors, antibacterial agents, etc., so as to introduce clear biological activities, such as ROS scavenging and anti-inflammation, etc., and thus, actively participate in the healing process [79].

#### Eliminating ROS and alleviating oxidative stress

Given that ROS plays a central role in the occurrence and progression of RISI, eliminating free radicals is an important therapeutic target. Second-generation hydrogels can encapsulate antioxidants such as gallic acid, epigallocatechin gallate (EGCG), curcumin, fullerenes, silica and cerium oxide [80]. For example, hydrogels containing aspirin have been shown to eliminate ROS and repair DNA double-strand breaks [81]. A drug-free antioxidant hydrogel can continuously exert antioxidant activity through the oxidation of disulfide bonds, thereby alleviating local oxidative stress and preventing RISI deterioration [71]. Hydrogels combining a catalytic antioxidant module (such as nanozyme or catechol-bearing network) with an NF-κB/TLR pathway modulator can synergize to shorten the high-ROS/high-cytokine window, thereby priming the wound bed for angiogenesis [82].

#### Targeting specific inflammatory mediators

Persistent inflammation is a key factor in the progression of RISI. Second-generation hydrogels can serve as carriers for anti-inflammatory drugs, such as curcumin, deferoxamine, retinoic acid and corticosteroids. For example, chondroitin sulfate can help control inflammation [83]. DNAzyme hydrogels have been shown to specifically inhibit the NLRP3 pathway, thereby alleviating the inflammatory response [29]. Additionally, the regulation of macrophage polarization towards the M2 phenotype (promoting healing) by glucan is also a potential target [84].

#### Promoting angiogenesis and improving microcirculation

Radiation-induced vascular injury is the main cause of RISI’s chronicity [24]. Second-generation hydrogel can encapsulate angiogenic factors (such as VEGF) or angiogenesis-promoting drugs (such as EGCG, Deferoxamine, Sildenafil). For example, VEGF- chitosan nanoparticles can protect endothelial cells and promote angiogenesis [85]. Self-assembling hydrogels combined with Deferoxamine can promote the formation of capillary-like structures and the growth of new blood vessels [86].

#### Promoting cell proliferation, migration and tissue regeneration

RISI is characterized by damaged skin cells (keratinocytes, fibroblasts) and impaired regeneration capacity [87]. Second-generation hydrogels can not only provide a scaffold to support cell growth and migration but also encapsulate growth factors (such as EGF, PDGF), peptides or keratin to promote epidermal tissue regeneration, fibroblast proliferation and epithelialization [88]. Mesenchymal stem cells (MSCs) and their exosomes have also been shown to promote epithelialization, enhance angiogenesis and inhibit fibrosis [89].

#### Interfering with pro-fibrotic signals

Second-generation hydrogels can deliver drugs or cells that inhibit skin fibrosis, such as adipose-derived stem cells (ADSCs) which have been shown to inhibit irregular collagen deposition [90]. Second-generation hydrogels can also provide local delivery of TGF- β inhibitors or drugs that regulate fibroblast activity [91].

#### Restoring skin microbiome homeostasis

Severe RISI is prone to secondary infections. Second-generation hydrogels can incorporate antimicrobials, such as chitosan, sodium alginate, graphene oxide or silver ions [92]. Second-generation hydrogels also can deliver probiotics, prebiotics or postbiotics to combat radiation-induced microbial dysbiosis.

Besides, benefiting from the application of advanced analysis tools, the treatment mode for RISI has transformed from targeting widespread inflammation to precisely locating specific immune signaling pathways (such as the IL-6/CCR6 axis, IL-17 signaling pathway, NLRP3 inflammasome) and intercellular interactions [20]. This shift requires the development of next-generation hydrogels capable of precise, localized and sustained delivery of highly specific immunomodulators (such as monoclonal antibodies, specific kinase inhibitors, therapeutic nucleic acids like Dnases).

However, the active ingredients encapsulated in 2G hydrogels (such as growth factors, enzymes) may have issues with poor stability and limited skin permeability. Despite improvements, some 2G hydrogels may still have insufficient mechanical strength, especially in parts that need to withstand mechanical stress. In addition, the extraction, purification and encapsulation of active biomaterials may lead to increased production costs. Moreover, certain components or high concentrations of drugs may pose potential risks of immunogenicity or cytotoxicity [93].

### Third generation hydrogels: smart responsive systems

Third generation hydrogels represent the forefront of hydrogel technology, designed to have smart responsiveness capable of sensing changes in the wound microenvironment and responding accordingly, meeting the need for temporally matched therapeutic intervention by utilizing quantifiable RISI biomarkers (pH, ROS, enzymes) as triggering stimuli [71, 94]. The RISI microenvironment exhibits marked spatiotemporal heterogeneity including acidic pH (6.5–7.0 vs. physiological 7.4), elevated ROS (100–500 μM H_2_O_2_) and upregulated proteases such as matrix metalloproteinases MMP-2 and MMP-9 [95]. Smart responsive hydrogels can accurately release therapeutic agents based on specific signals from the wound microenvironment or external stimuli, providing a more adaptable treatment platform to fit the multivariable complexity of different healing stages [96]. Some smart responsive hydrogels can also integrate sensing components for continuous monitoring of biomarkers such as pH value, glucose or inflammatory markers in wound exudate, providing real-time information on wound status and enabling personalized wound management [97].

Representative examples demonstrate the clinical potential of these systems. Xia *et al*. [71] reported an X-ray responsive drug-free antioxidant hydrogel formed by copolymerization of disulfide-containing hyperbranched polymers that exhibit continuous antioxidant activity through oxidation of disulfide bonds and triggered release of growth factors after radiation-responsive network breakage, showing efficacy in preventing radiation skin injury deterioration. Zhou *et al*. [98] designed a DNAzyme hydrogel targeting NLRP3 overexpression, where DNAzyme was encapsulated in ZIF-8 nanoparticles connected to TAT transmembrane peptide to enhance transdermal permeability. This DZ hydrogel effectively inhibited NLRP3 expression *in vitro* and promoted wound healing in RISI mouse models by regulating apoptosis, oxidative stress and inflammatory response proteins.

**Figure 3:**
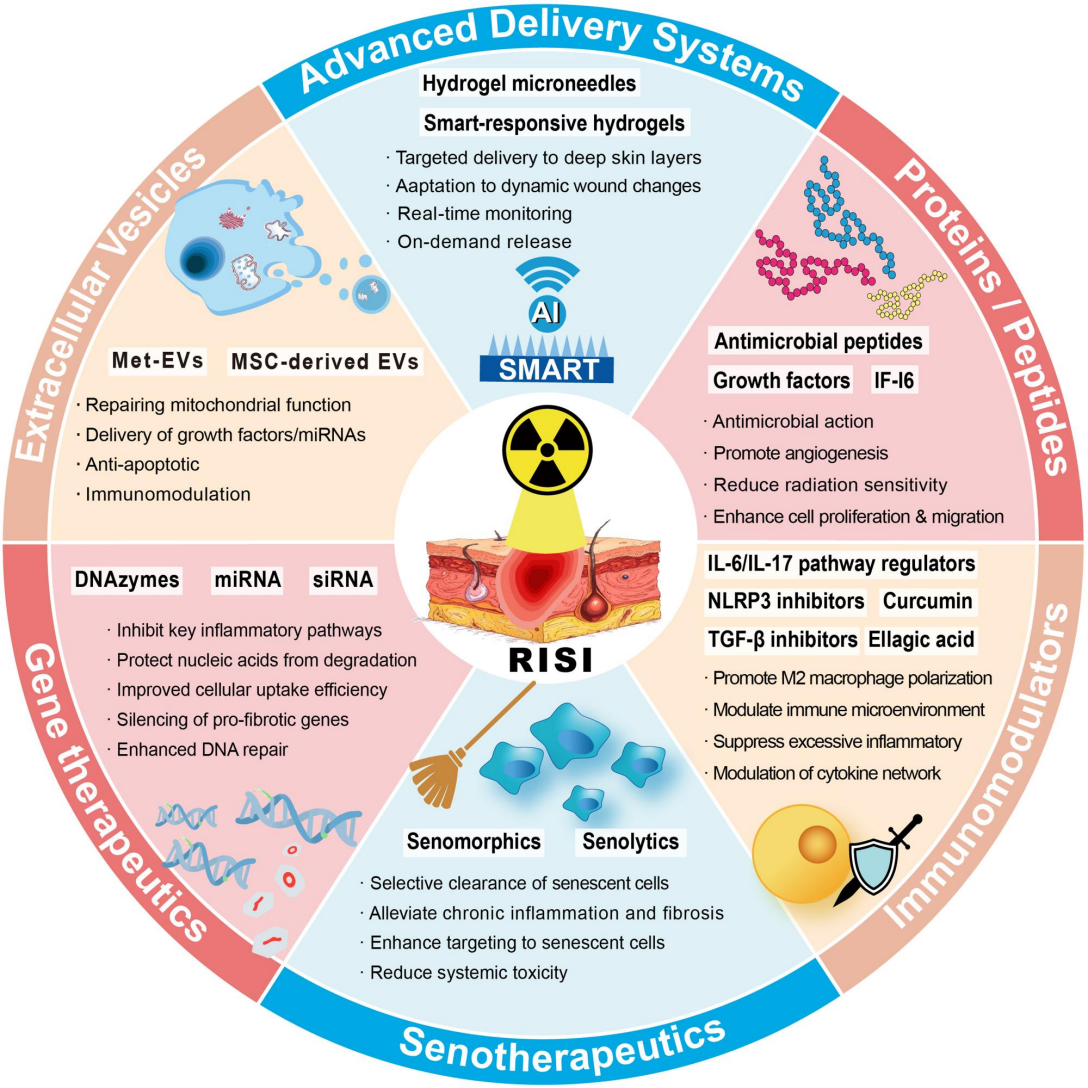
Advanced therapeutic payloads: from small molecules to living cells. RISI, radiation-induced skin injury; EVs, extracellular vesicles; Met-EVs, mitochondria-enriched EVs; MSC, mesenchymal stem cells; AI, artificial intelligence.

**Figure 4:**
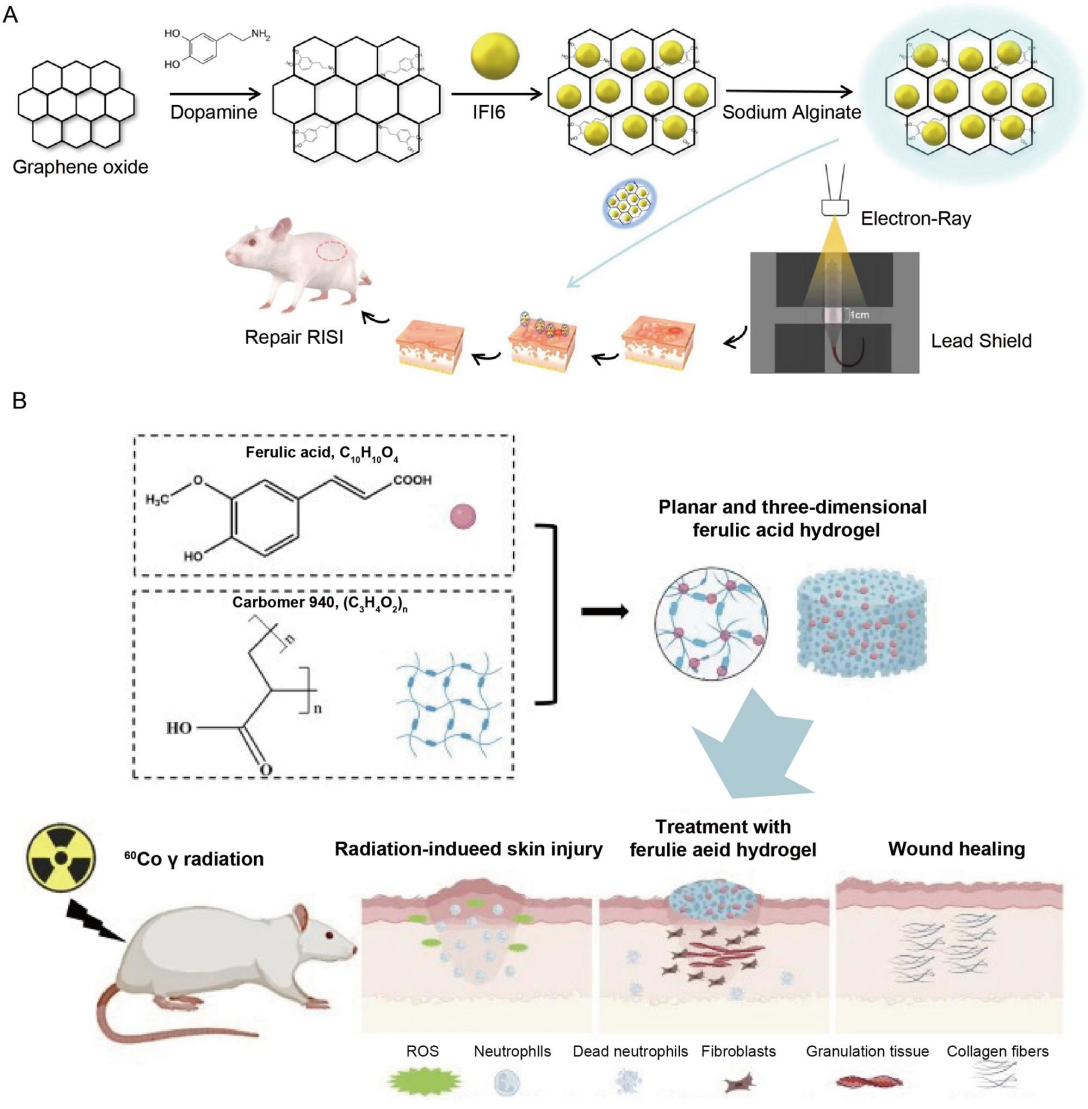
Recent progress of advanced hydrogel formulations for RISI (Part 1). (**A**) the fabrication of IFI6-PDA@GO/SA hydrogel and its application for RISI healing. With approval, reprinted from Ref. [134]. Copyright © 2022, BioMed Central Ltd. (**B**) The design route and application of FA-based carbomer hydrogel. With approval, reprinted from Ref. [136]. Copyright © 2024, BioMed Central Ltd.

**Figure 5:**
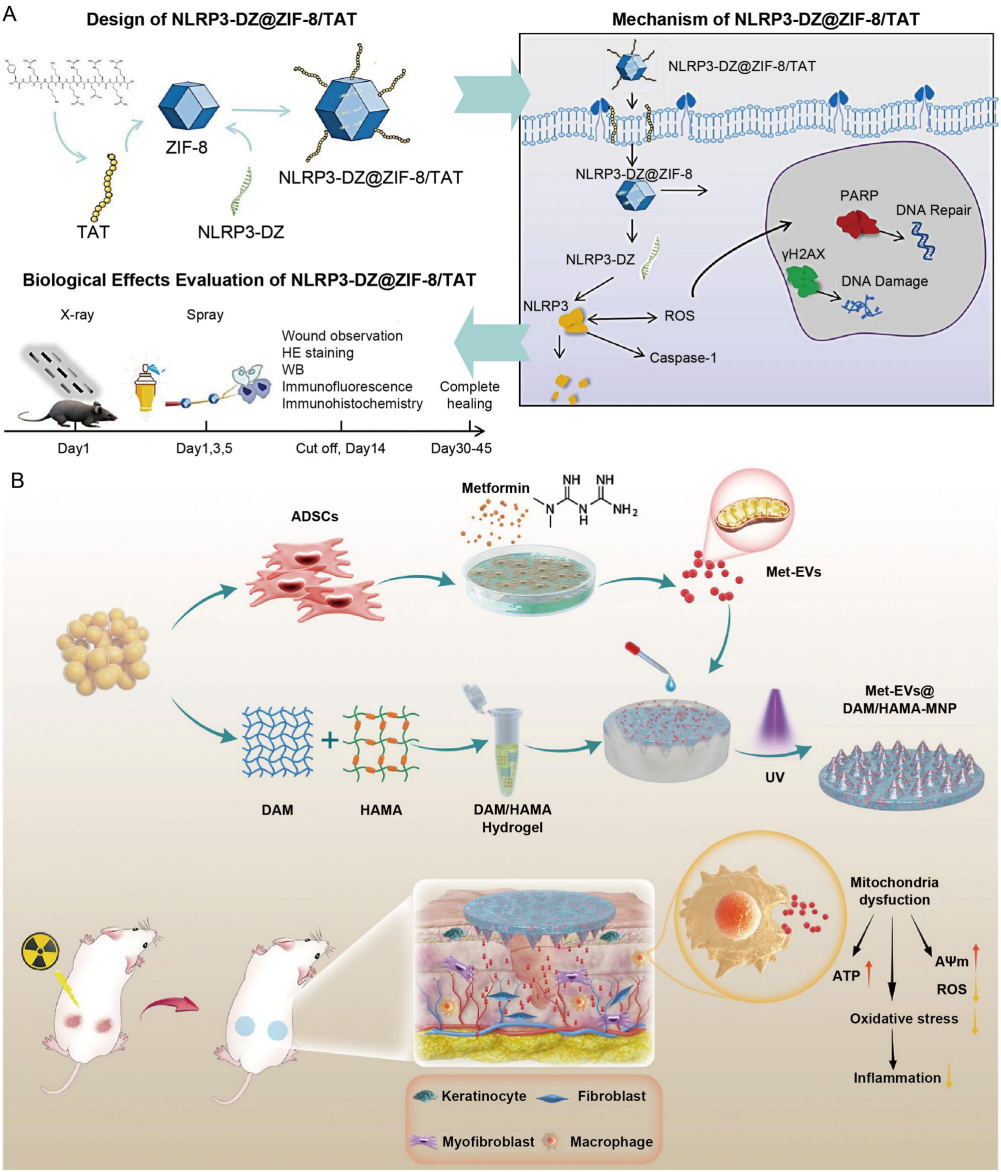
Recent progress of advanced hydrogel formulations for RISI (Part 2). (**A**) the design route and research of NLRP3-DZ@ZIF-8/TAT DNAzyme Hydrogel. With approval, reprinted from Ref. [98]. Copyright © 2025, BioMed Central Ltd. (**B**) The fabrication of Met-EVs@DAM/HAMA-MNP and skin wound treatment in a mice model. With approval, reprinted from Ref. [137]. Copyright © 2024, American Chemical Society.

**Figure 6:**
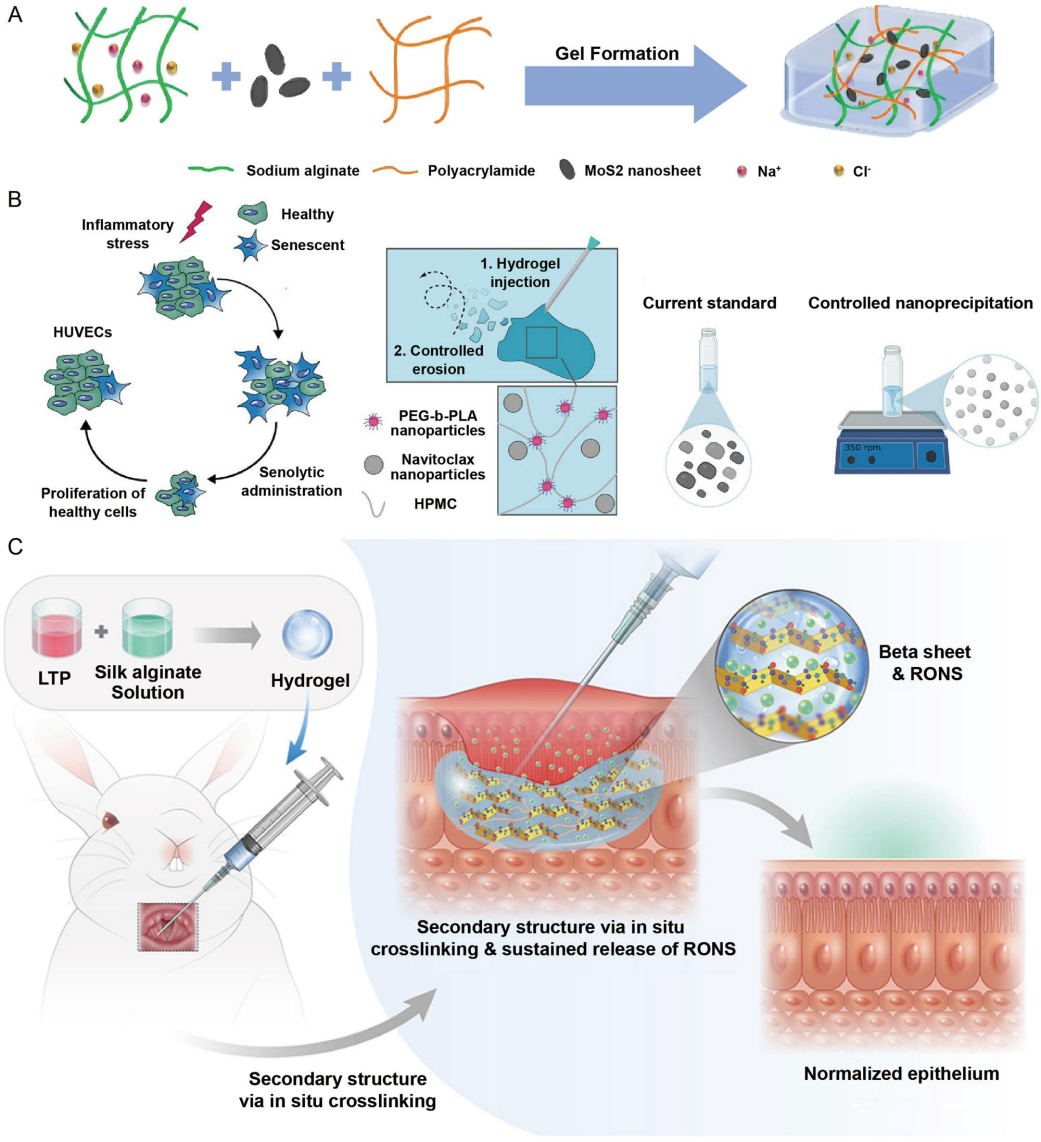
Recent progress of advanced hydrogel formulations for RISI (Part 3). (**A**) the design route double network hydrogel doped with MoS_2_ nanosheets. With approval, reprinted from Ref. [119]. Copyright © Royal Society of Chemistry 2024. (**B**) Local injectable hydrogel loaded with controlled navitoclax nanoparticles. With approval, reprinted from Ref. [130]. Copyright © 2025, American Chemical Society. (**C**) The design route and application of injectable silk-alginate crosslinking composite hydrogel. With approval, reprinted from Ref. [138]. Copyright © 2022 Elsevier B.V. HPMC, hydroxypropyl methylcellulose; PEG-b-PLA, polyethylene glycol block polylactic acid; HUVECs, human umbilical vein endothelial cells; RONS, reactive oxygen and nitrogen species.

However, the design and synthesis of smart responsive hydrogels are more complex, requiring precise material selection and crosslinking mechanisms. For example, some electrically responsive hydrogel systems traditionally rely on redox reactions for actuation, raising concerns over material safety; leveraging synergistic noncovalent interactions such as host–guest electrostatic repulsion and hydrogen bonding represents a safer and more controllable alternative [99]. Additionally, traditional smart hydrogels may have weak mechanical properties and slow response times, although methods such as constructing reversible noncovalent bonds can enhance their mechanical properties and self-healing ability [100]. The development of hydrogels does not have clear generational differences. Based on the generational classification in current mainstream research, we have divided hydrogels into these three categories. Currently, hydrogels that integrate new effective payloads and delivery methods are still widely used in RISI treatment research (Table 3 and Figures 3–6). Table 3 summarizes representative advanced hydrogel formulations for RISI and their key components/payloads (e.g. IFI6‑PDA@GO/SA, FA‑based carbomer, NLRP3‑DZ@ZIF‑8/TAT, Met‑EVs@DAM/HAMA‑MNP, MoS_2_-doped double-network, navitoclax‑nanoparticle injectable depot and LTP‑induced silk–alginate composite hydrogels). Figure 3 schematically summarizes advanced therapeutic payloads from small molecules to living cells, whereas Figures 4–6 compile the corresponding representative formulations in Parts 1–3.

**Table 3:** Recent progress of advanced hydrogel formulations for RISI.

Hydrogel type	Main components	Key characteristic	Mechanism	Application effect/Potential	Ref.
IFI6-PDA@GO/SA Hydrogel	IFI6 protein, PDA, GO, SA	Reduce radiation sensitivity, have antibacterial and anti ROS properties, promote cell migration and vascularization, inhibit apoptosis, improve immune microenvironment	Modulate immune response by activating the SBP1/HSF1 signaling pathway and reducing ROS/NLRP3 expression	IFI6 was used for RISI treatment for the first time, capable of accelerating RISI healing, reducing healing time and improving the immune microenvironment in mouse models, which showed a clinical application potential.	[134]
FA- based carbomer hydrogel	Carbomer 940, FA	Good biocompatibility, antioxidant capacity, reduces inflammation and oxidative stress, accelerates tissue reconstruction and collagen deposition	Inhibit NLRP3 inflammasome activation	Significant recovery effect on radiation-induced skin damage has been demonstrated both *in vitro* and *in vivo*.	[136]
NLRP3-DZ@ZIF-8/TAT DNAzyme Hydrogel	DZ targeting NLRP3, ZIF-8 nanoparticles, TAT transmembrane peptide	High biological safety, inhibit NLRP3 expression, promote cell migration, inhibit cell apoptosis, antibacterial properties, Enhance transdermal penetration	Specifically inhibit the NLRP3 pathway, regulate proteins related to apoptosis, oxidative stress and inflammatory response	Good results were observed in both *in vitro* cell models and RISI mouse models. New strategy for the prevention and treatment of RISI.	[98]
Met-EVs@DAM/ HAMA-MNP	DAM/HAMA hydrogel microneedles, Metformin handled Met- EVs	Release Met EVs and their mitochondria, alleviate mitochondrial dysfunction, promote macrophage polarization towards M2 type, enhance healing	Transfer active mitochondria to rescue mitochondrial membrane potential, increase ATP and reduce ROS	Superior effect on promoting wound healing was demonstrated in X-ray induced mitochondrial dysfunction model. Potential clinical value for chronic wounds.	[137]
Double network hydrogel doped with MoS_2_ nanosheets	PAM, SA nanofibers, MoS_2_ nanosheets	Good mechanical and adhesive properties, without secondary dressing fixation, remove ROS and reduce oxidative stress	Reduce oxidative damage through the antioxidant properties endowed by MoS_2_ nanosheets	The biosafety assessment showed that it had practicality and potential as an external dressing for skin radiation protection.	[119]
Navitoclax Nanoparticle Polymer Hydrogel	HPMC, PEG-b-PLA, Navitoclax	Injectable senescent cell scavenger reservoir, locally deliver Navitoclax, selectively eliminate senescent cells	Induce apoptosis of senescent cells through Navitoclax as an inhibitor of the Bcl-2 family; release drugs by the erosion of hydrogels	*In vitro* experiments showed that it could eliminate senescent endothelial cells and restore cell proliferation effectively. Potential application value for RISI for the same pathological mechanism.	[130]
Silk-alginate crosslinking composite hydrogel	SF, SA, LTP	Controlled release of bioactive effectors and enhanced mechanical properties	LTP-induced silk crosslinking; calcium ion crosslinking of alginate; sustained biochemical effects of reactive oxygen and nitrogen species.	The *in vitro* and *in vivo* results showed that it could reduce scar formation, promote favorable wound healing, increase ECM deposition and improve its distribution, thus, achieving functional repair.	[138]

IFI6, interferon alpha inducible protein 6; PDA, polydopamine; GO, graphene oxide; DZ, DNAzyme; ZIF-8, zeolitic imidazolate framework-8; TAT, trans-activator of transcription; DAM, decellularized adipose-derived matrix; HAMA, hyaluronic acid methacrylate; FA, ferulic acid; PAM, polyacrylamide; SF, Silk Fibroin; SA, sodium alginate; LTP, liquid-type nonthermal atmospheric plasma; HPMC, hydroxypropyl methylcellulose; PEG-b-PLA, polyethylene glycol.

#### Stimuli-responsive hydrogels: rationale and design principles

The RISI microenvironment exhibits marked spatiotemporal heterogeneity in pathological stimulus that static drug-delivery systems cannot adequately address. Stimuli-responsive hydrogels exploit these endogenous disease biomarkers as triggers for autonomous therapeutic responses, achieving unprecedented precision in matching treatment to real-time wound status. For instance, self-assembled (supramolecular) peptide-based hydrogels offer a versatile, sequence-programmable platform with tunable microstructural and mechanical properties [101]. Key pathological stimuli include: (a) pH deviations from physiological neutrality. RISI lesions can exhibit acidic pH (6.5–7.0) in inflamed and hypoxic regions, whereas nonhealing/chronic wounds may display broader pH heterogeneity. Accordingly, pH should be viewed as a stage-dependent and spatially heterogeneous trigger, rather than a unidirectional ‘acidic-to-7.4 normalization’ signal [102]. Beyond serving as hydrogel triggers, the combination of acidic pH and H_2_O_2_ can also be exploited for intra-biofilm delivery; chemically modified and UV-inactivated bacteria have been shown to selectively integrate into same-species biofilms and release antibiotics in response to local pH and hydrogen peroxide, providing a conceptual template for biofilm-penetrating designs [103]. (b) Elevated ROS (100–500 μM H_2_O_2_) during acute oxidative burst (Days 0–7 post-radiation), persisting at lower levels (50–100 μM) in chronic wounds beyond 4 weeks, sourced from neutrophil NADPH oxidase, mitochondrial dysfunction and radiation-generated hydroxyl radicals [71]. (c) Upregulated proteases, particularly matrix metalloproteinases MMP-2 and MMP-9 elevated 5- to 10-fold in chronic RISI driving extracellular matrix degradation and delayed re-epithelialization [104]. (d) Hypoxia (<5% O_2_, ∼38 mmHg) due to radiation-induced vascular damage reducing capillary density by 40–60%, limiting oxygen supply critical for collagen synthesis and keratinocyte proliferation [105].

Design principles across stimuli-responsive platforms emphasize: (a) Response kinetics matching pathological timescales, requiring rapid ROS scavenging within hours during acute phase versus sustained anti-fibrotic release over weeks in chronic phase. (b) Selectivity to distinguish pathological from physiological signals, for example differentiating ROS elevation during normal macrophage-mediated pathogen clearance (transient 200–400 μM H_2_O_2_ bursts) from chronic inflammation (sustained 50–150 μM). (c) Reversibility enables repeated, on-demand release and allows pH-responsive hydrogels to re-seal as the local pH normalizes; it is often strengthened by modular self-assembling peptide or peptide-amphiphile designs that integrate bioactive epitopes, enzyme-cleavable sequences and redox- or pH-labile linkers [106]. (d) Biocompatibility of trigger-responsive moieties and their degradation byproducts, ensuring that diselenide bond oxidation products do not induce cytotoxicity [107]. Data-driven/AI-assisted approaches are also accelerating the discovery and optimization of self-assembling peptide building blocks.

ROS-responsive hydrogels represent a rational design strategy targeting persistent oxidative stress characteristic of RISI. These systems incorporate chemical bonds undergoing oxidation-triggered cleavage, such as thioketal linkers, phenylboronic acid esters and diselenide bonds [108]. For instance, Wang *et al*. [109] developed a ROS/pH dual-responsive hydrogel loaded with curcumin nano-micelles, demonstrating 68.75% drug release under combined acidic and high ROS conditions compared to 41.34% under physiological conditions, enabling targeted therapy in infected radiation wounds. However, emerging evidence suggests that ROS-responsive hydrogels should extend beyond passive antioxidant delivery to active immunomodulation. Recent nanocatalytic strategies have revealed that controlled ROS generation—rather than elimination—can orchestrate immune responses. Piezoelectric-enhanced nanocatalysts disrupt redox homeostasis to trigger neutrophil N1 polarization against bacterial biofilms [110], while bandgap-engineered germanene nanosheets (direct bandgap 1.65 eV) enable photodynamic singlet oxygen (^1^O_2_) generation with favorable biodegradability [111]. Notably, oxygen-immunomodulated nanocatalysts exploit light-activated ^1^O_2_ to sequentially create transient hypoxia (prolonging neutrophil lifespan) and subsequent reoxygenation (triggering neutrophil ‘immune switch’ for enhanced NETosis and phagocytosis), achieving superior anti-infection efficacy through trained immunity [110]. These findings suggest a paradigm shift for RISI hydrogels: integrating photosensitizers or piezoelectric materials to achieve on-demand, spatiotemporally controlled ROS generation synchronized with immune activation, balancing oxidative stress elimination with therapeutic immune programming.

pH-responsive hydrogels exploit the acidic microenvironment characteristic of radiation-damaged tissue. The pH gradient from 6.5–7.0 in damaged tissue to physiological 7.4 in healed wounds provides a temporal biomarker that normalizes during healing progression [112]. pH-responsive hydrogels typically incorporate ionizable polymer groups such as carboxyl moieties in poly(acrylic acid) or alginate that deprotonate at pH values above their pKa causing electrostatic repulsion, chain extension, hydrogel swelling and drug release. Swelling ratios can increase substantially as pH shifts from acidic to physiological values, enabling controlled drug payload delivery [113]. Alternatively, cationic polymers like chitosan protonate at acidic pH triggering different conformational changes. This pH-sensitivity can be exploited for phase-matched therapy, with rapid anti-inflammatory drug release during acute phases followed by sustained pro-angiogenic factor delivery during proliferative phases. However, modest pH changes in RISI wounds (pH 6.5–7.0) require highly sensitive polymers and buffering capacity of tissue fluids may dampen responses. Multi-stimuli systems combining pH-sensitivity with ROS or enzyme responsiveness offer improved selectivity through AND-gate logic gates [114].

Enzyme-responsive hydrogels target dramatic proteolytic dysregulation characteristic of RISI, where matrix metalloproteinases particularly MMP-2 and MMP-9 are substantially elevated in chronic injuries. These zinc-dependent endopeptidases degrade collagen and gelatin, impairing extracellular matrix integrity. Enzyme-responsive hydrogels incorporate peptide sequences such as GPQG-IWGQ (cleavage site between glutamine and isoleucine) serving as crosslinkers or drug-conjugate linkers highly selective for MMP-2 and MMP-9 [115]. When these proteases cleave the peptide backbone, network degradation triggers drug release with kinetics proportional to enzyme concentration. Alternative substrates such as PVGLIG offer broader MMP specificity, while neutrophil elastase-cleavable sequences like AAPV enable targeting of acute inflammatory phases characterized by neutrophil infiltration. Dual-enzyme logic gates requiring both MMP and elastase cleavage provide enhanced specificity, with complete network degradation occurring only in severe RISI exhibiting concurrent protease elevation [116]. Zhao *et al*. [117] reported an ultrafast enzyme-responsive hydrogel for real-time assessment and treatment optimization in infected wounds, where MMP-2-triggered drug release enabled dynamic therapeutic adjustments based on wound enzyme profiles.

DNAzyme-based hydrogels represent an emerging frontier in precision RISI therapy by targeting specific inflammatory pathways at the molecular level. Zhou *et al*. [98] designed a DNAzyme hydrogel specifically inhibiting the NLRP3 inflammasome pathway to prevent RISI in mice. DNAzyme was encapsulated in zeolitic imidazolate framework-8 (ZIF-8) nanoparticles conjugated to TAT transmembrane peptide to enhance transdermal permeability. This DZ hydrogel effectively inhibited NLRP3 expression *in vitro* and promoted wound healing in RISI mouse models by regulating apoptosis, oxidative stress and inflammatory response proteins. Genomic analysis revealed that DNAzyme treatment significantly downregulated genes associated with inflammatory response and cytokine production while upregulating genes involved in wound healing and tissue regeneration. This approach overcomes limitations of traditional small-molecule drugs through catalytic nucleic acid activity enabling sustained therapeutic effects without payload depletion.

Multi-stimuli responsive hydrogels integrate two or more environmental triggers to achieve Boolean logic-gate control over drug release, enhancing therapeutic precision. Dual ROS/pH responsive systems exhibit synergistic release kinetics, where drug liberation occurs only when both oxidative stress and acidosis exceed pathological thresholds, a signature combination distinguishing chronic RISI from acute-phase inflammation or normal wound healing. Triple-responsive platforms incorporating temperature sensitivity enable external modulation of drug release through localized mild hyperthermia (40–42°C), providing clinician-controlled triggering complementing autonomous microenvironment responses [118]. However, increased design complexity raises translational challenges including reproducible manufacturing, regulatory approval complexity for combination products and potential for unintended cross-reactivity between stimuli-responsive moieties.

Translational challenges confronting stimuli-responsive hydrogels include: (a) Specificity limitations and a narrow safety window, as ROS levels spike during physiological healing when macrophage-mediated pathogen clearance generates transient H_2_O_2_ bursts essential for antimicrobial defense; overly sensitive ROS scavenging and trigger-coupled immunomodulation may blunt protective inflammation (infection risk) and, if mistimed, contribute to aberrant remodeling, including fibrosis and hypertrophic scarring[68]; (b) Incomplete therapeutic coverage stemming from limited scavenging capacity, as many hydrogels exhibit saturation after consuming limited ROS equivalents, necessitating incorporation of catalytic antioxidants such as cerium oxide nanoparticles or Prussian blue nanozymes with continuous redox cycling capabilities [119]. In addition to ROS scavenging, emerging piezoelectric-enhanced nanocatalysts can disrupt redox homeostasis to trigger neutrophil N1 polarization against bacterial biofilms, supporting a materials-driven immunomodulation paradigm for infection control [120]. (c) Spatial heterogeneity within RISI wounds where hydrogen peroxide concentrations vary substantially from necrotic wound centers to proliferative edges to peri-wound skin causing uneven drug distribution, potentially addressable through integrated real-time biosensors enabling closed-loop feedback control [121]. (d) Regulatory complexity as combination products containing biomaterials and drugs require dual regulatory oversight, manufacturing scalability challenges in reproducing stimuli-responsive chemistries with batch-to-batch consistency at commercial scale and clinical validation requiring demonstration of superiority over standard-of-care in adequately powered randomized controlled trials with clinically meaningful endpoints such as time to complete wound closure and prevention of Grades 3–4 progression [122].

#### Advanced therapeutic payloads: from small molecules to living cells

Beyond smart responsive mechanisms, 3G hydrogels distinguish themselves through sophisticated cargo integration encompassing biologics previously incompatible with conventional delivery systems. The synergy between stimuli-responsive matrices and advanced therapeutic payloads enables precision medicine approaches where the *what* (therapeutic agent), *when* (triggered release timing) and *where* (spatial localization) are simultaneously optimized.

Cell-based therapies and extracellular vesicles (EVs) represent the most complex biologics integrated into hydrogel platforms. MSCs and their secreted EVs including exosomes possess potent regenerative and immunoregulatory capabilities through paracrine release of growth factors, cytokines and microRNAs that promote angiogenesis, suppress inflammation and mitigate apoptosis and fibrosis [123]. Hydrogels function as three-dimensional niches preserving cell viability and sustaining EV bioactivity by protecting against enzymatic degradation and enabling controlled spatiotemporal release [124]. Met-EVs@DAM/HAMA-MNP hydrogel microneedle patches exemplify this approach, delivering mitochondria-enriched EVs that restore mitochondrial function and polarize macrophages toward the M2 phenotype, accelerating healing of radiation-combined injuries [125]. The hydrogel scaffold not only protects fragile EVs from immune clearance but also enables penetration through the stratum corneum barrier via microneedle geometry, addressing a major limitation in topical EV delivery. Beyond MSC-derived EVs, probiotics-derived bacterial EVs are also attracting growing interest for skin repair and regeneration and hydrogel integration can further improve their local retention and delivery efficiency [126].

Gene therapy modalities including plasmid DNA, small interfering RNA (siRNA) and microRNA (miRNA) provide molecular-level intervention targeting pathogenic gene expression programs in RISI. Hydrogel-mediated nucleic acid delivery overcomes traditional barriers including nuclease degradation, poor cellular uptake and off-target distribution. For instance, siRNA silencing pro-fibrotic genes such as transforming growth factor-β or collagen type I alpha 1 delivered via cationic polymer hydrogels can attenuate RIF progression, while pro-angiogenic miRNAs such as miR-181a packaged in hydrogel nanocarriers enhance vascular regeneration [127]. DNAzyme hydrogels targeting the NLRP3 inflammasome represent a particularly innovative approach, combining the structural functions of hydrogel matrices with catalytic nucleic acid activity to directly modulate inflammatory signaling pathways, achieving sustained therapeutic effects without cargo depletion [98]. The key advantage of hydrogel gene delivery lies in localized, sustained nucleic acid presentation to target cells, minimizing systemic exposure and associated toxicities while maintaining therapeutic concentrations throughout the prolonged RISI healing timeline spanning weeks to months.

Senolytic and senomorphic agents address the fundamental pathobiology of chronic RISI driven by accumulation of senescent cells secreting senescence-associated secretory phenotype (SASP) factors that perpetuate inflammation and fibrosis. Senolytics such as Bcl-2 inhibitors selectively induce apoptosis of senescent cells, while senomorphics suppress SASP without direct cytotoxicity [128]. These agents offer mechanistic approaches to breaking the self-sustaining inflammatory loops characteristic of chronic radiation injury [129]. Navitoclax, a Bcl-2 family inhibitor, when formulated as nanoparticles and encapsulated in polymer nanoparticle (PNP) hydrogels, creates an on-demand reservoir enabling sustained senescent cell clearance [130]. Although the original study targeted wound healing rather than RISI specifically, the mechanism directly addresses radiation-induced senescent cell accumulation driving chronic pathology including dermal fibrosis and persistent inflammation documented in preclinical RISI models. Hydrogel delivery is particularly advantageous for senolytics given their potential systemic toxicities, as localized sustained release confines drug exposure to injured tissue while maintaining therapeutic concentrations sufficient for senescent cell elimination over treatment durations of weeks.

Immunomodulatory agents targeting specific immune cell subpopulations or inflammatory signaling cascades represent rational therapeutic interventions for RISI characterized by dysregulated immunity. Strategies include promoting regulatory T cell expansion, driving M2 macrophage polarization toward pro-regenerative phenotypes [131] or blocking pro-inflammatory pathways such as NLRP3 inflammasome, transforming growth factor-β, interleukin-6 and interleukin-17 signaling [132]. Immunomodulatory hydrogel patches loaded with nanoparticles assembled from curcumin and tannic acid exemplify multifunctional platforms combining anti-inflammatory, antioxidant and immune-regulatory activities for radiation dermatitis treatment and radioprotection [98]. The hydrogel matrix enables sustained release kinetics matching the prolonged inflammatory phase of RISI while the nanoparticle formulation enhances cellular uptake and intracellular delivery of hydrophobic immunomodulators.

Advanced antioxidant systems beyond conventional small-molecule ROS scavengers incorporate nanomaterial-based catalytic antioxidants with sustained activity. Novel platforms include MoS_2_ nanosheets with intrinsic peroxidase-like activity catalyzing hydrogen peroxide decomposition [119], graphene-based materials functionalized with redox-active moieties providing continuous ROS neutralization [133] and enzyme-mimetic nanoparticles such as cerium oxide exhibiting regenerative antioxidant cycling. These nanomaterial-hydrogel composites overcome limitations of traditional antioxidants including rapid depletion, inability to catalytically regenerate and poor retention at injury sites. The hydrogel serves dual functions: dispersing nanomaterials to maximize reactive surface area and preventing nanomaterial aggregation that would diminish catalytic efficiency.

Other bioactive molecules encompassing peptides, recombinant proteins and natural product extracts expand the therapeutic arsenal. Interferon alpha-inducible protein 6 (IFI6) promotes keratinocyte survival and migration under radiation stress [134], while botanicals such as soy isoflavone extracts provide anti-inflammatory and antioxidant activities [98] and cannabidiol exhibits immunomodulatory effects reducing pro-inflammatory cytokine production [45]. Hydrogel encapsulation stabilizes these diverse agents against degradation while enabling combination therapy approaches where multiple bioactives with complementary mechanisms are co-delivered.

The integration of advanced therapeutic payloads into smart responsive hydrogels represents a paradigm shift from simple drug depots to intelligent therapeutic systems. However, translational challenges include: maintaining bioactivity of labile biologics during hydrogel fabrication processes involving crosslinking chemistries or thermal treatments; achieving clinically relevant dosing for expensive biologics such as growth factors or nucleic acids within practical hydrogel volumes; regulatory complexities for combination products containing multiple therapeutic modalities; and demonstrating added clinical value justifying increased complexity and cost compared to standard-of-care treatments [135].

## Enabling technologies and clinical translation

The translation of advanced hydrogel therapeutics from bench to bedside requires convergence of molecular profiling technologies, computational design tools and innovative manufacturing platforms. In parallel, scalable biomimetic immunotherapeutics are emerging; for example, a redox-responsive natural killer cell mimic has been developed to eliminate intracellular pathogen infections, highlighting a complementary direction for hard-to-reach intracellular reservoirs relevant to complicated wound settings [139]. Single-cell RNA sequencing has revealed cellular heterogeneity and dysregulated intercellular communication networks underlying chronic RISI, providing molecular rationales for precision therapeutic targeting [20, 28, 140]. Artificial intelligence is accelerating materials discovery by predicting structure–property relationships from compositional inputs while enabling patient stratification through predictive modeling of radiation dermatitis risk [141]. Three-dimensional bioprinting technologies enable fabrication of hydrogel constructs with precise spatial control over architecture and bioactive agent distribution [135]. Advanced delivery platforms including dissolving microneedle arrays overcome stratum corneum barrier limitations [103, 25], while evolved preclinical models employing fractionated irradiation regimens and large animal systems improve clinical translatability [136, 142]. Despite these advances, substantial barriers including regulatory complexity, manufacturing scalability and health economics considerations must be addressed to realize the clinical potential of smart responsive hydrogels.

### Enabling technologies for precision hydrogel engineering

**Figure 7:**
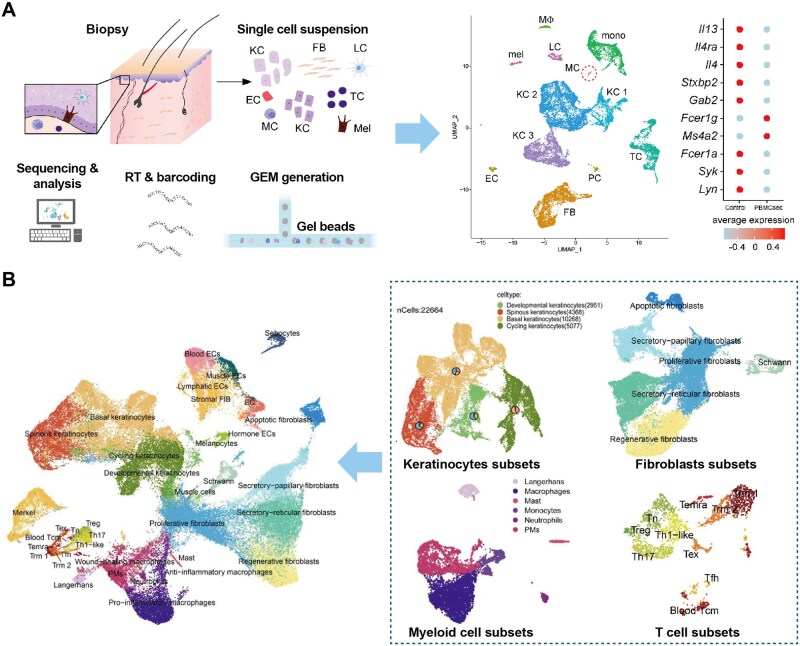
Application of single-cell sequencing in RISI mechanism and hydrogel design. (**A**) Experimental approach of single cell RNA sequencing: Uniform manifold approximation and projection (UMAP) plot of sec- and medium-treated skin and expression of mast cell-specific genes. With approval, reprinted from Ref. [144]. Each dot represents one cell, color code indicates identified cell clusters. Copyright © 2022, Elsevier B.V. (**B**) UMAP visualization of the cellular diversity in RISI and the characterization of keratinocytes subsets, fibroblasts subsets, myeloid cell subsets and T cell subsets in RISI. With approval, reprinted from Ref. [145]. Copyright © 2025, BioMed Central Ltd. EC, endothelial cells; FB, fibroblasts; KC1, keratinocyte cluster 1; KC2, keratinocyte cluster 2; KC3, keratinocyte cluster 3; LC, Langerhans cells; MC, mast cells; mel, melanocytes; mono, monocytes; MP, macrophages; PC, pericytes; TC, T cells.

Single-cell sequencing technologies have revolutionized our understanding of RISI pathobiology by revealing cellular heterogeneity and dysregulated intercellular communication networks previously obscured by bulk tissue analysis [143]. Paldor *et al*. [28] identified a senescence-associated IL-6/CCR6 axis driving chronic radiodermatitis, where senescent keratinocytes secreting interleukin-6 recruit CCR6-positive inflammatory T cells, perpetuating a self-amplifying inflammatory loop. Yan *et al*. [20] performed comprehensive single-cell analysis using samples from radiation accident patients and irradiated rats, revealing that exposure to 30 Gy electron beam irradiation caused significant upregulation of fibroblasts and endothelial cells with downregulation of keratinocytes. Among five common differentially expressed genes between human and rat skin, Nur77 was highly expressed in fibroblasts and validated as a critical regulator of RISI. Laggner *et al*. [144] demonstrated through single-cell analysis of irradiated mouse skin that radiation fundamentally alters interaction intensities among fibroblasts, endothelial cells and dendritic cells, with universal upregulation of IL-17 signaling suggesting IL-17 serves as a central hub orchestrating RISI pathogenesis [140]. These molecular insights enable rational hydrogel design targeting specific cell populations and signaling axes. For instance, the discovery of IL-6/CCR6 axis provides rationale for hydrogels delivering IL-6 receptor antagonists or CCR6-blocking peptides, while identification of Nur77 upregulation in fibroblasts suggests incorporating Nur77 inhibitors to prevent fibrotic progression. Yu *et al*. [145] further performed molecular profiling of skin cells identifying distinct cellular signatures in RISI across various stages in murine datasets, revealing temporal dynamics of immune cell infiltration, keratinocyte dysfunction and fibroblast activation that inform stage-specific hydrogel therapeutic strategies (Figure 7).

Artificial intelligence and machine learning are accelerating hydrogel materials discovery and clinical outcome prediction [146]. Li *et al*. [147] comprehensively reviewed AI-energized design and optimization of hydrogels for biomedical applications, demonstrating that machine learning techniques including neural networks, random forests and support vector machines can predict structure-property relationships from compositional inputs, reducing experimental iterations significantly compared to traditional trial-and-error approaches. In materials discovery, AI algorithms trained on databases of polymer compositions, crosslinking densities and mechanical/swelling properties can predict optimal formulations for target specifications such as desired degradation kinetics or drug release profiles [27]. Importantly, data-driven and AI-assisted strategies are also beginning to accelerate the discovery and optimization of self-assembling peptide building blocks for supramolecular hydrogels [148]. Collectively, these computational approaches can shorten development timelines from year to month. For clinical translation, Lin *et al*. [149] developed machine learning models for predicting radiation dermatitis in breast cancer patients using clinical risk factors, patient-reported outcomes and serum cytokine biomarkers. Feature selection identified 18 predictors of Grade 2 or higher radiation dermatitis, with baseline levels of interleukin-1β, MMP-9 and vascular endothelial growth factor among the most predictive biomarkers. The XGBoost model achieved the highest performance with an area under the receiver operating characteristic curve of 0.780 (95% confidence interval: 0.701–0.854), enabling identification of high-risk patients who would benefit most from prophylactic hydrogel application. Lee *et al*. [150] applied ensemble machine learning methods including extreme gradient boosting to predict radiation dermatitis risk in head-neck cancer patients receiving proton therapy, with models achieving area under the curve (AUC) of 0.890, significantly outperforming conventional logistic regression (AUC 0.740). These predictive models enable personalized prevention strategies where hydrogel prophylaxis is deployed preemptively in high-risk individuals, shifting the paradigm from reactive treatment to proactive prevention. But these tools should be positioned as clinician decision support rather than autonomous controllers, with predefined override and escalation pathways. Safety-focused evaluation should explicitly consider failure modes (such as false negatives and distribution shift) to ensure that AI malfunction does not delay standard-of-care interventions.

These predictive models enable personalized prevention strategies where hydrogel prophylaxis is deployed preemptively in high-risk individuals, shifting the paradigm from reactive treatment to proactive prevention. However, these tools should be positioned as clinician decision support rather than autonomous controllers, with predefined override and escalation pathways. Safety-focused evaluation should explicitly consider failure modes (e.g. false negatives and distribution shift) to ensure that AI malfunction does not delay standard-of-care interventions and clinical evaluation/reporting should follow established guidance (e.g. SPIRIT-AI/CONSORT-AI; TRIPOD+AI), including external validation and calibration in addition to discrimination metrics [151–153]. Importantly, any AI-enabled or closed-loop workflow should be fail-safe—defaulting to standard-of-care with clinician override/escalation pathways and post-deployment monitoring—so that model drift or sensor/algorithm failure cannot delay timely intervention [154].

Three-dimensional bioprinting technologies enable fabrication of hydrogel constructs with precise spatial control over composition, architecture and bioactive agent distribution that is unattainable through conventional casting or molding methods [135]. Extrusion-based bioprinting deposits hydrogel bioinks layer-by-layer to create constructs mimicking native tissue architecture [155], enabling creation of bilayers or multilayer hydrogels where superficial layers contain antimicrobial agents for infection prevention while deeper layers release pro-angiogenic factors promoting vascular regeneration [156]. Stereolithography and digital light processing techniques offer higher resolution (10–50 μm feature sizes) enabling micropatterning of adhesive ligands or growth factor gradients that guide cellular behaviors including migration directionality and differentiation [157]. Patient-specific hydrogel patches matching individual wound geometries can be manufactured using 3D scanning of injury sites coupled with computer-aided design, ensuring optimal contact and drug delivery across irregular wound surfaces common in RISI [158].

**Figure 8:**
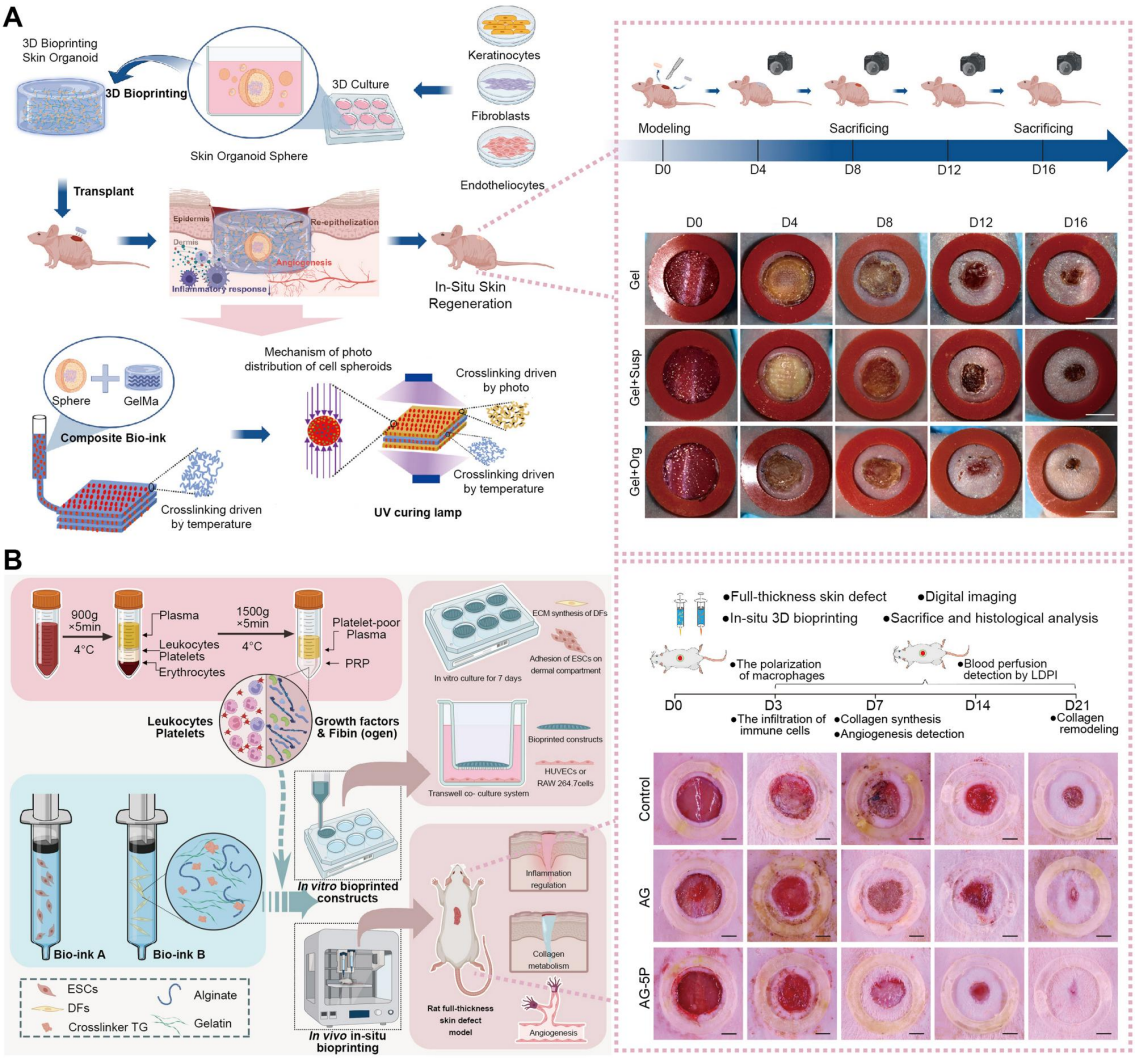
Application of 3D bioprinting in hydrogel dressing. (**A**) A gel material combining skin organoids and 3D bioprinting technology. With approval, reprinted from Ref. [159]. Copyright © 2024, Elsevier B.V. on behalf of KeAi communications Co. Ltd. The schematic diagram illustrates the preparation of 3D bioprinting skin organoids developed from three adult stem cells for the restoration of large skin defects *in situ* and improved healing quality. The general observation of wound treated with different dressing was photted by digital camera in every 4 days. The rubber ring was an anti-shrinkage ring with an inner diameter of 1 cm. (**B**) A 3D bioprinting technology with PRP-integrated AG composite hydrogel. With approval, reprinted from Ref. [160]. Copyright © 2022, Elsevier Ltd. The schematic diagram illustrates the bioprinting process using PRP containing multicomponent bio-ink. The general observation of wound treated with different dressing was photographed by digital camera. The rubber ring was an anti-shrinkage ring with an inner diameter of 1 cm. PRP, platelet-rich plasma; AG, alginate-gelatin; ESCs, epidermal stem cells; DFs, dermal fibroblasts.

However, translational challenges include: limited availability of bioinks that are mechanically robust for high-mobility or high-tension anatomical sites while maintaining cytocompatibility; printing speed limitations that hinder on-demand manufacturing in acute care settings; and evolving regulatory pathways for patient-customized medical devices (Figure 8). In addition, translation of patient-specific bioprinting requires predefined critical quality attributes, including print fidelity, sterility assurance, residual crosslinker/initiator limits, mechanical acceptance windows and batch-to-batch reproducibility, together with explicit discussion of manufacturing failure risks. Trial reporting should transparently document device versioning, human factors and on-site manufacturing variability to avoid idealized descriptions [151].

Advanced delivery platforms including microneedle arrays and wearable biosensor-integrated systems represent innovations in hydrogel application methodology rather than hydrogel chemistry itself, yet profoundly impact therapeutic efficacy. Dissolving microneedle patches composed of biocompatible polymers such as hyaluronic acid or polyvinylpyrrolidone enable transdermal delivery of hydrogel-encapsulated therapeutics, bypassing the stratum corneum barrier that limits topical drug penetration to typically <5% of applied dose [161]. Microneedles create temporary microchannels (50–900 μm depth) through which hydrogel formulations containing macromolecular drugs, nanoparticles or biologics can access viable epidermis and dermis where pathological processes occur. Ma *et al.* developed a PEG-SOD-loaded dissolving microneedle (PSMN) patch for the prevention of radiation dermatitis. By bypassing the stratum corneum barrier, the PSMN patch achieved a 500-fold increase in drug skin permeation compared to topical PEG-SOD solution, significantly improving radioprotection efficacy [162]. Wearable hydrogel sensors integrating electrochemical or optical detection elements enable continuous monitoring of wound biomarkers such as pH, temperature, matrix metalloproteinase activity or inflammatory cytokine levels, providing real-time feedback on healing status and enabling closed-loop therapeutic systems where drug release is automatically triggered when biomarkers exceed pathological thresholds [163]. However, challenges include: patient acceptance of microneedle applications particularly in pediatric or elderly populations; potential for microchannel-mediated bacterial invasion requiring incorporation of antimicrobials; and technical complexity of integrating biosensors with controlled-release hydrogels while maintaining sensor accuracy and hydrogel biocompatibility.

**Figure 9:**
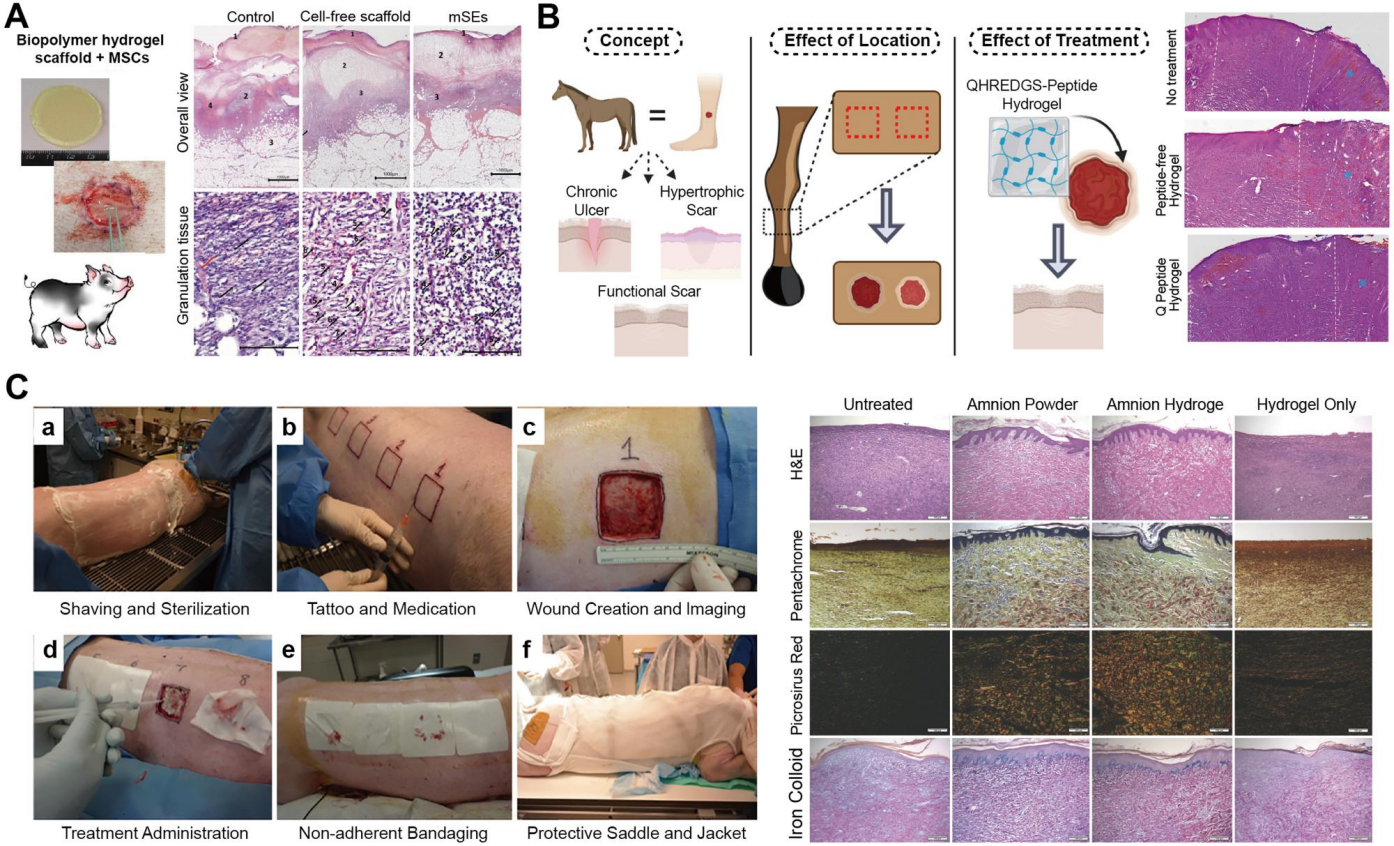
The validation of hydrogel materials in large experimental animals. (**A**) A biopolymer hydrogel scaffold with encapsulated MSCs. With approval, reprinted from Ref. [168]. Copyright © 2025, MDPI (Basel, Switzerland). Histological view shows the comparation of control wound, model cell-free scaffold wound and model skin equivalents (mSEs) wound on Day 7. (**B**) Effect of location and treatment with a peptide-modified collagen–chitosan hydrogel in an equine limb model. With approval, reprinted from Ref. [169]. Copyright © 2020, American Chemical Society. Histological view shows the representative 20× images from Day 28 biopsies cryosection (35 μm). (**C**) Amnion membrane hydrogel and amnion membrane powder in a full thickness porcine skin wound model. With approval, reprinted from Ref. [170]. Copyright © 2019, John Wiley & Sons, Inc. Histological view shows the representative images of H&E, pentachrome, picrosirius red and iron colloid staining.

Preclinical models for RISI hydrogel evaluation have evolved from simple acute radiation dermatitis models toward more clinically relevant systems [164]. Traditional models using single-fraction high-dose irradiation (20–30 Gy) on rodent skin induce acute ulceration within 2–3 weeks but fail to recapitulate the chronic fibrosis and vascular damage characteristic of fractionated clinical radiotherapy [142]. Advanced models employing fractionated irradiation regimens (e.g. 2 Gy × 30 fractions) better mimic clinical scenarios, exhibiting delayed fibrosis onset (8–12 weeks post-irradiation) and persistent inflammation. Large animal models including minipigs offer skin anatomy and healing kinetics more similar to humans compared to rodents, with epidermal thickness and dermal-epidermal junction architecture resembling human skin [136, 165]. *Ex vivo* human skin models using full-thickness skin explants maintained in organ culture following irradiation enable testing hydrogel therapeutics on human tissue while reducing animal use and potentially improving clinical translatability [166]. However, *ex vivo* models lack systemic circulation and immune components, limiting evaluation of hydrogels modulating systemic inflammation or recruiting circulating stem cells [167]. Radiation-combined injury models incorporating burn, infection or wound co-insults better represent clinical scenarios where radiotherapy patients may develop opportunistic infections or mechanical injury to compromised skin [125]. Despite advances, no preclinical model fully recapitulates human RISI pathophysiology, necessitating validation across multiple model systems and ultimately clinical trials for definitive efficacy demonstrations (Figure 9).

**Figure 10:**
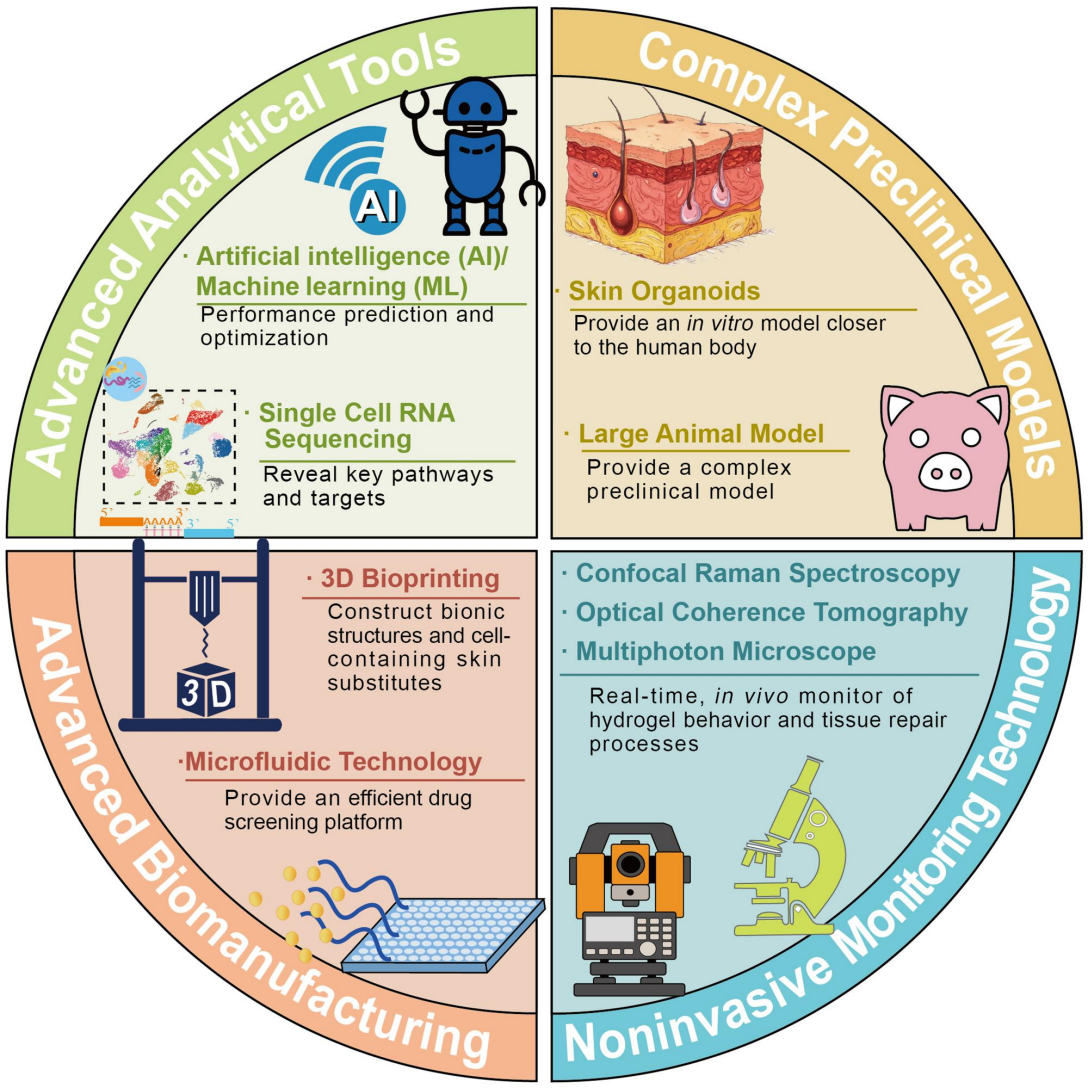
Interdisciplinary innovation and technology for RISI hydrogel therapy.

In addition, microfluidic technology is used to prepare structurally uniform, size-controllable microgels, microspheres or microfluidic chips with complex structures for drug screening or constructing *in vitro* RISI models [171]. Skin organoids derived from pluripotent stem cells or adult stem cells can better simulate the complex structure (epidermis, dermis, hair follicles and sebaceous glands) and physiological functions of human skin [172], providing a more human-like *in vitro* model for studying the mechanisms of RISI and testing the efficacy of hydrogels. Skin organoids can also be used for drug screening, evaluating the biocompatibility of hydrogels, improving drug release efficiency and promoting the regeneration of damaged skin [173]. By combining advanced imaging technologies such as confocal Raman spectroscopy (CRS), optical coherence tomography (OCT) and multiphoton microscopy, it is possible to noninvasively monitor the degradation behavior, drug release, collagen deposition and angiogenesis of hydrogels in the RISI model, thus, providing dynamic efficacy assessment data [174] (Figure 10).

### Clinical translation: from bench to bedside

#### Current clinical landscape and unmet needs

Beyond acute erythema and moist desquamation, late toxicities such as fibrosis, telangiectasia and lymphedema substantially impair quality of life in RISI patients. RISI remains highly prevalent among breast [175] and head-and-neck cancer [176] patients due to large irradiated surface areas, skin folds and moisture and concurrent systemic therapies. Current hydrogel products used for RISI management primarily provide moist wound environments, exudate absorption and pain relief through passive mechanisms. However, clinical evidence reveals significant limitations of these conventional approaches. A randomized controlled trial comparing hydrogel dressings with dry dressings for treating moist desquamation demonstrated significantly prolonged healing time in the hydrogel group with no significant impact on patients’ subjective skin symptoms [177]. This paradoxical finding suggests that moisture retention alone may be insufficient or even counterproductive without addressing underlying pathological mechanisms including persistent inflammation, oxidative stress and impaired angiogenesis.

Analysis of clinical trial data (Table 4) reveals inconsistent outcomes across different hydrogel formulations. While aloe vera-based gel reduced dermatitis severity and delayed onset in head-and-neck cancer patients [178], liposomal gel with chamomile showed no statistically significant differences compared to controls [179]. Film-forming silicone gels such as StrataXRT demonstrated reduced physiological skin damage parameters [180], yet head-to-head comparison with Mepitel film showed nuanced superiority depending on endpoint [181]. These discrepancies underscore the need for mechanistically informed hydrogel design rather than empirical formulation optimization. Notably, multifunctional and smart-responsive hydrogel products that have reached market launch or entered late-stage clinical research remain scarce, with most innovations confined to preclinical stages [185].

**Table 4:** Several gel-based materials and related drugs under clinical trial for RISI treatment.

Intervention	Cancer	Study design	*n*	Primary EPs	Key results	Ref.
Aloe vera-based gel	Head & Neck	Phase III multicenter RCT, double-blind, placebo-controlled	120	CTCAE grade; symptoms	Aloe vera gel reduced severity and delayed onset of dermatitis. There was no prophylactic efficacy for RID.	[178]
Liposomal gel with chamomile	Head & Neck	Randomized, controlled (parallel)	60	CTCAE grade; pain; QoL	No statistically significant differences in outcomes. Chamomile liposomal gel showed better prevention signals.	[179]
Film-forming silicone gel (StrataXRT)	Breast	Prospective clinical study (controlled)	56	Objective skin biophysiology, CTCAE	StrataXRT reduced physiological skin damage parameters and dermatitis severity vs control group with X-derm.	[180]
Mepitel film vs. StrataXRT gel	Breast	Randomized head-to-head	99	Incidence of ≥G2 dermatitis	Differences between Mepitel film and StrataXRT were explored, with nuanced superiority depending on endpoint.	[181]
Topical curcumin (turmeric) gel	Mixed (breast focus)	Phase Ⅱrandomized RCT, placebo-controlled	52	CTCAE grade; pain/itch	2% Curcumin gel in reducing skin side effects during breast cancer radiation therapy	[182]
Poly-herbal gel	Head & Neck	Prospective randomized	71	CTCAE grade; pruritus pain VAS	Preventive herbal gel alleviated dermatitis-related symptoms and reduced grade incidence	[183]
Episil topical use for irradiated skin	Breast	Single-Center, Open, Parallel, Phase I/II RCT	102	CTCAE grade; pain; time to recovery	Episil improved acute radiation dermatitis scores and symptom relief vs control Expanded off-label skin use requires confirmation	[184]

StrataXRT, silicone film-forming gel dressing; Mepitel film, elastic soft silicone film; Episil, bio-adhesive barrier-forming gel used for oral mucosal; X-derm, moisturizing cream; EPs, endpoints; CTCAE, common terminology criteria for adverse events; RID, radiation induced dermatitis; RCT, randomized controlled trial; QoL, quality of life; VAS, visual analog scale.

Despite these trials, the clinical evidence for hydrogel interventions in RISI remains difficult to interpret due to pronounced heterogeneity in patient populations (cancer site, concurrent therapy), radiotherapy techniques, intervention intent, outcome definitions and comparators (usual care, placebo or film dressings). Many studies are small, single-center or open-label, which increases performance and assessment bias—particularly for subjective endpoints such as pain, pruritus and ‘time to recovery’. Therefore, future translation should avoid class-wide claims for ‘hydrogels’ and instead match dressing physicochemical properties (moisture balance, exudate absorption, antimicrobial capacity) to RISI stage and lesion severity, while adopting standardized endpoints and blinded assessment where feasible.

Prevention-focused strategies offer promise in shifting the paradigm from reactive treatment to proactive protection. Certain hydrogels containing high atomic number elements or high-density materials can provide radioprotection by absorbing or scattering portions of radiation dose [186]. A Phase III randomized double-blind placebo-controlled trial demonstrated that boron-based hydrogel containing 3% sodium pentaborate pentahydrate significantly reduced incidence of dermatitis, erythema, dry desquamation and moist desquamation in breast cancer radiotherapy patients [187]. Similarly, prophylactic application of FLAMIGEL significantly reduced moist desquamation incidence and delayed its onset [177]. However, preventive efficacy exhibits substantial individual variability influenced by anatomical site, radiation dose fractionation and patient-specific factors including skin phototype and baseline inflammatory status. Optimal timing and protocols for prophylactic hydrogel application remain undefined, requiring integration with predictive biomarkers to identify high-risk patients who would derive maximum benefit.

#### Regulatory pathways and manufacturing scalability

Regulatory classification critically determines the translational pathway and timeline for hydrogel-based RISI therapeutics. Hydrogels functioning solely as physical barriers or wound dressings without pharmacological claims may qualify as Class II medical devices under FDA 510(k) clearance requiring demonstration of substantial equivalence to predicate devices—a relatively streamlined pathway achievable within 6–12 months. However, hydrogels incorporating drugs, biologics or claims of treating specific pathological processes typically require classification as combination products overseen by FDA’s Office of Combination Products, necessitating Investigational New Drug (IND) applications and potentially full New Drug Application (NDA) with randomized controlled trials [137]. The regulatory burden is compounded for multicomponent systems such as stimuli-responsive hydrogels containing nanoparticles, where each component’s safety profile must be independently established and potential synergistic toxicities evaluated. Harmonization of regulatory requirements across jurisdictions (FDA, EMA, PMDA) remains incomplete, hindering global development strategies. Recent guidance documents including FDA’s ‘Guidance for Industry: Expedited Programs for Serious Conditions’ offer accelerated pathways such as Fast Track designation, breakthrough therapy designation and priority review for products addressing unmet medical needs in severe RISI, potentially reducing approval timelines by 6–12 months [138].

Manufacturing scalability poses substantial challenges transitioning from laboratory-scale batch synthesis to current Good Manufacturing Practice (cGMP) production. Although many hydrogel components possess good biocompatibility, long-term *in vivo* safety and potential immunogenicity of complex multifunctional hydrogels (particularly those containing nanoparticles, stimuli-responsive components and bioactive molecules) require rigorous evaluation [21]. Establishing robust quality control assays for hydrogels is complicated by their three-dimensional network structure and water content (typically 80–99% water) which precludes many standard analytical techniques. Critical quality attributes requiring validation include: degree of crosslinking affecting mechanical properties and degradation rates; homogeneity of drug or nanoparticle distribution preventing ‘hot spots’ or ‘cold spots’ in therapeutic delivery; sterility assurance particularly for hydrogels incorporating biologics such as growth factors or cells; and stability during storage, as many hydrogels undergo syneresis (spontaneous water expulsion) or gradual crosslink degradation over time. For degradable hydrogels, biocompatibility of degradation products must be assessed and degradation rates must match tissue repair/regeneration rates [188, 189]—rapid degradation may cause premature therapeutic agent release and insufficient structural support, while slow degradation may hinder tissue integration or trigger foreign body reactions. Continuous manufacturing approaches replacing traditional batch processes offer improved process control and reduced contamination risks but require substantial capital investment and regulatory validation [190].

#### Clinical validation and health economics

Clinical trial design for hydrogel therapeutics confronts multiple methodological challenges stemming from RISI heterogeneity. Clinical performance and severity of RISI are influenced by numerous factors including radiotherapy dose, anatomical site and individual patient differences, complicating endpoint evaluation. Primary endpoints in RISI trials commonly include: time to complete wound healing (defined as 100% re-epithelialization maintained for 2 weeks without dressing requirement); proportion of patients avoiding progression to Grade 3 radiation dermatitis (confluent moist desquamation); reduction in pain scores assessed via visual analog scales or Patient-Reported Outcomes Measurement Information System (PROMIS) pain interference instruments; and quality-of-life measures using validated instruments such as Skindex-16 or Dermatology Life Quality Index [144]. Challenges in endpoint selection include variability in RISI severity grading systems (CTCAE vs. RTOG vs. LENT-SOMA scales exhibiting only moderate inter-rater agreement, *κ* = 0.6–0.7) and subjective components of pain assessment. Appropriate comparator selection remains debated: placebo-controlled trials maximize internal validity but may be ethically problematic in severe RISI where standard-of-care treatments exist, while active-controlled noninferiority designs require large sample sizes (typically 300–500 patients per arm) to demonstrate noninferiority margins clinically meaningful to justify the innovation [145]. Adaptive trial designs employing interim analyses with pre-specified decision rules for early stopping or dose modification offer efficiency advantages but require sophisticated statistical planning.

Standardization of preclinical evaluation is essential to improve clinical translatability. Currently, hydrogel research requires establishing more standardized preclinical animal models closely mimicking clinical scenarios and optimizing *in vitro* testing methods to improve predictability of preclinical research results [191]. Development and validation of biomarkers capable of objectively assessing RISI severity, predicting treatment responses and monitoring healing processes are essential to guide clinical trials and personalized treatment. Candidate biomarkers include serum inflammatory cytokines (interleukin-1β, MMP-9, vascular endothelial growth factor) demonstrated to predict Grade 2+ radiation dermatitis with AUC of 0.780 [149] and imaging-based assessment of dermal blood flow and collagen density using OCT or multiphoton microscopy [174].

Health economics and market access increasingly influence translation success. While advanced hydrogel therapies involve higher upfront costs, their clinical value lies in preventing progression to severe Grades 3–4 RISI that necessitate surgical intervention, treatment interruptions and prolonged hospitalization—outcomes that impose substantial burden on both patients and healthcare systems [192]. However, translating preclinical efficacy into real-world clinical benefit requires demonstration of effectiveness across diverse patient populations beyond controlled trial settings. Clinical adoption challenges include heterogeneous healthcare infrastructure limiting access in resource-limited settings, variable insurance coverage policies that may classify hydrogels as ‘wound care supplies’ rather than therapeutic agents and insufficient clinical education regarding patient selection criteria and application protocols (Table 5).

**Table 5:** RISI hydrogel clinical translation: status, prevention methods and main challenges.

Aspect	Clinical status	Challenges	Strategy
Therapeutic hydrogel	Clinically, traditional hydrogels are predominantly employed.	Inconsistent clinical trial results are inconsistent. Lackness of clinical data of advanced intelligent hydrogel. Biocompatibility and long-term safety. Complex clinical trial design.	Develop advanced hydrogels based on pathological mechanism. Standardize preclinical evaluation. Develop objective biomarkers. Adopt adaptive clinical trial design.
Preventive hydrogel	Boron-based hydrogels and silicone gel dressings have already been applied.	Individual differences in preventive efficacy. Optimal timing and protocols. Persistence in long-term prevention.	Prevent precisely targeting high-risk populations. Develop more efficient and convenient preventive hydrogel formulations. Integrate AI prediction models to guide prevention.
Production and supervision	Current production is limited to the laboratory, and there is a limited capacity for large-scale GMP production.	The expanded application of the production process of complex hydrogels Quality control and batch-to-batch consistency. Unclear regulatory pathways for novel combination products.	Develop modular and automated production processes. Communication with regulatory agencies early. Establish industry standards.
Cost and accessibility	The R&D costs for advanced hydrogels are high.	Failure to achieve superior cost-effectiveness compared to current treatments. Challenges with health insurance coverage.	Perform comprehensive health economic assessments. Optimize production processes for cost reduction.

GMP, Good Manufacturing Practice; R&D, research and development.

#### Future roadmap: toward precision medicine

Closed-loop theranostic systems integrating multiple enabling technologies promise transformative advances beyond current passive delivery paradigms. Wearable hydrogel sensors incorporating electrochemical or optical detection elements enable continuous monitoring of wound biomarkers such as pH, temperature, matrix metalloproteinase activity or inflammatory cytokine levels, providing real-time feedback on healing status [163]. When coupled with electronically triggered drug release mechanisms controlled via smartphone applications [137], these systems enable precision dosing adjusted in real-time to individual healing trajectories. For instance, detection of pH elevation above 7.5 (indicating bacterial infection) or MMP-9 concentration exceeding 50 ng/mL (indicating excessive inflammation) could automatically trigger release of antimicrobials or anti-inflammatory agents from stimuli-responsive nanocarriers embedded within the hydrogel matrix.

Artificial intelligence integration extends beyond materials discovery to clinical decision support. AI algorithms analyzing wound images captured via smartphone cameras combined with biomarker data could predict healing outcomes and recommend therapeutic adjustments, extending specialist dermatology expertise to under-resourced settings [137]. Machine learning models achieving AUC of 0.890 for radiation dermatitis prediction in head-neck cancer patients [150] demonstrate feasibility of this approach. Integration of such predictive models with AI-optimized hydrogel formulations [147] creates an adaptive therapeutic ecosystem where patient-specific risk profiles guide both preventive and treatment strategies.

**Figure 11:**
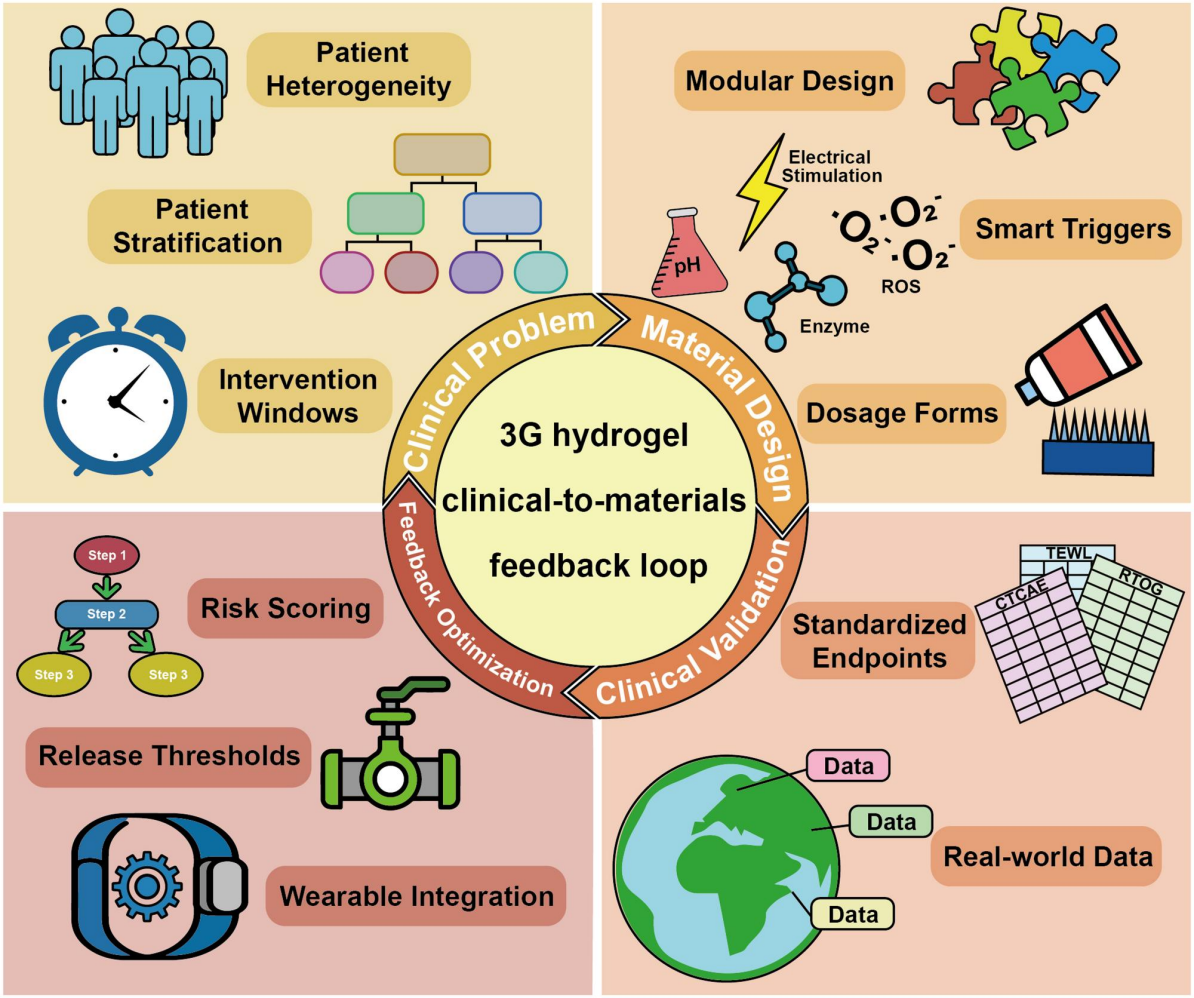
A clinical-to-materials feedback loop for RISI hydrogels. Clinical heterogeneity drives modular design and implementable formats. Standardized endpoints and real-world validation feedback to refine stratification and release logic.

Personalized bioprinting represents the ultimate convergence of precision medicine and advanced manufacturing. Bioprinted hydrogels incorporating patient-derived induced pluripotent stem cells differentiated into keratinocytes and fibroblasts could provide autologous cell therapy overcoming immune rejection risks. Three-dimensional bioprinting technologies enable fabrication of bilayer constructs where superficial layers contain antimicrobial agents while deeper layers release pro-angiogenic factors [193], with stereolithography achieving 10–50 μm resolution for micropatterning adhesive ligands [157]. Patient-specific hydrogel patches matching individual wound geometries can be manufactured using 3D scanning of injury sites coupled with computer-aided design, ensuring optimal contact and drug delivery across irregular wound surfaces common in RISI [194]. However, regulatory pathways for such personalized medical devices remain undefined, requiring individual device approval that may be logistically infeasible for widespread clinical adoption (Figure 11).

Realizing this vision requires sustained interdisciplinary collaboration among materials scientists, radiation oncologists, dermatologists, regulatory scientists and health economists, coupled with funding mechanisms supporting translational research bridging the ‘valley of death’ between promising technologies and clinical products. Establishment of consortia such as the Radiation Dermatitis Collaborative working group fostering data sharing, protocol harmonization and multicenter trials will accelerate the field toward evidence-based precision medicine for RISI. Early communication with regulatory agencies regarding approval requirements for novel combination products and personalized devices will facilitate smooth clinical translation [195]. Ultimately, success will be measured not by technological sophistication alone, but by demonstrable improvement in patient-centered outcomes including reduced pain, accelerated healing, prevention of chronic sequelae and enhanced quality of life for the millions of cancer survivors affected by RISI.

## Summary

Evolving from the first generation of passive dressings that provide a moist healing environment, through the second generation of active biomaterials capable of delivering drugs and cells, to the third generation of systems with intelligent response and bioprinting capabilities, hydrogel technology has made significant progress in RISI management. These advancements enable hydrogels to target the key pathological mechanisms of RISI, including oxidative stress, inflammation, vascular injury and fibrosis, thereby promoting more comprehensive tissue repair and regeneration. Mechanotransduction-based strategies may further broaden this toolbox; chemical substrate-enabled piezoelectric therapy has been reported to integrate antibiofilm action with macrophage metabolic reprogramming to break chronic-wound feedback loops, providing an instructive paradigm for multimodal design [196]. Future hydrogels will focus more on personalization, with customization based on the patient’s specific type of injury, severity, genetic background and healing ability [197], including developing hydrogels that can integrate the patient’s autologous cells (such as stem cells), platelet-rich plasma (PRP) enriched with growth factors or exosomes to promote more effective tissue regeneration and repair [198].

The development of hydrogels capable of performing multiple functions simultaneously represents an important trend. For example, smart hydrogels that integrate biosensors (for real-time monitoring of the wound microenvironment’s pH, ROS, glucose or inflammatory markers), drug delivery (on-demand release of therapeutic agents), antibacterial and regeneration-promoting functions represent an extremely considerable development potential [199, 200]. These systems will be able to dynamically adjust treatment strategies according to the different stages of wound healing.

Three-dimensional bioprinting technology will continue to drive the application of hydrogels in RISI treatment. By precisely depositing cells and bio-inks, skin substitutes with complex multilayer structures and vascularization can be constructed to more accurately simulate the physiological function of natural skin [201]. Future research will focus on overcoming the challenges of cell viability, resolution and vascularization in bioprinting [74]. Exploration and research will place greater emphasis on exploring new natural and synthetic polymers with enhanced mechanical properties, controllable biodegradability, better biocompatibility and stronger therapeutic effects [202]. At the same time, the milder and more efficient crosslinking methods will also be developed to maintain the activity and function of encapsulated cells and bioactive molecules [203].

By combining big data analysis and artificial intelligence, researchers can better understand the complex pathological mechanisms of RISI, predict patient responses to specific hydrogel treatments and optimize the design and treatment plans of hydrogels. Additionally, the development of hydrogel dressings integrated into wearable devices will achieve the goal of remote and continuous monitoring of the wound healing process, providing more convenient care for patients and real-time treatment feedback for clinicians [204, 205].

Although hydrogels show great transformative potential in the treatment of RISI, their clinical translation still faces numerous challenges, including strict regulatory approval, manufacturing scalability, mechanical properties and long-term safety issues, as well as the need for further accumulation of clinical evidence. Future development will focus on the integration of personalized medicine, multifunctional smart hydrogel systems, 3D bioprinting and tissue engineering, as well as innovations in new biomaterials and crosslinking technologies. By overcoming these challenges and fully leveraging emerging technologies, hydrogel materials are expected to fundamentally transform the clinical management of RISI, significantly improve patient prognosis and quality of life and ultimately optimize the overall effectiveness of cancer treatment.

## References

[1] Hu Y , Yu L, Du W, Hu X, Shen Y. Global hotspots and research trends of radiation-induced skin injury: a bibliometric analysis from 2004 to 2023. Front Oncol 2024;14:1430802. 39252945 10.3389/fonc.2024.1430802 PMC11381223

[2] Liu Z , Gu J, Gao Y, Hu H, Jiang H. ADSC-derived exosomes mitigate radiation-induced skin injury by reducing oxidative stress, inflammation and cell death. Front Public Health 2025;13:1603431. 40438040 10.3389/fpubh.2025.1603431 PMC12116344

[3] Yang X , Ren H, Guo X, Hu C, Fu J. Radiation-induced skin injury: pathogenesis, treatment, and management. Aging (Albany NY) 2020;12:23379–93. 33202382 10.18632/aging.103932 PMC7746368

[4] Rosenthal A , Israilevich R, Moy R. Management of acute radiation dermatitis: a review of the literature and proposal for treatment algorithm. J Am Acad Dermatol 2019;81:558–67. 30802561 10.1016/j.jaad.2019.02.047

[5] Ervin MD , Goans R, Diffenderfer-Stewart K, Aloisi B, Iddins CJ. Cutaneous radiation injuries: REAC/TS clinical experience. Disaster Med Public Health Prep 2024;18:e33. 38384188 10.1017/dmp.2023.233

[6] Wang Y , Liu H, He Y, Li M, Gao J, Han Z, Zhou J, Li J. Hydrogel-based strategies for the prevention and treatment of radiation-induced skin injury: progress and mechanistic insights. Biomimetics (Basel) 2025;10:758. 41294430 10.3390/biomimetics10110758 PMC12649924

[7] Fang Z , Lv Y, Zhang H, He Y, Gao H, Chen C, Wang D, Chen P, Tang S, Li J, Qiu Z, Shi X, Chen L, Yang J, Chen X. A multifunctional hydrogel loaded with two nanoagents improves the pathological microenvironment associated with radiation combined with skin wounds. Acta Biomater 2023;159:111–27. 36736645 10.1016/j.actbio.2023.01.052

[8] Wei J , Meng L, Hou X, Qu C, Wang B, Xin Y, Jiang X. Radiation-induced skin reactions: mechanism and treatment. Cancer Manag Res 2019;11:167–77. 30613164 10.2147/CMAR.S188655 PMC6306060

[9] Asadi N , Pazoki-Toroudi H, Del Bakhshayesh AR, Akbarzadeh A, Davaran S, Annabi N. Multifunctional hydrogels for wound healing: special focus on biomacromolecular based hydrogels. Int J Biol Macromol 2021;170:728–50. 33387543 10.1016/j.ijbiomac.2020.12.202

[10] Ho T-C , Chang C-C, Chan H-P, Chung T-W, Shu C-W, Chuang K-P, Duh T-H, Yang M-H, Tyan Y-C. Hydrogels: properties and applications in biomedicine. Molecules 2022;27:2902. 35566251 10.3390/molecules27092902 PMC9104731

[11] Qi L , Zhang C, Wang B, Yin J, Yan S. Progress in hydrogels for skin wound repair. Macromol Biosci 2022;22:e2100475. 35388605 10.1002/mabi.202100475

[12] Hao Y , Li H, Guo J, Wang D, Zhang J, Liu J, Yang C, Zhang Y, Li G, Liu J. Bio-inspired antioxidant heparin-mimetic peptide hydrogel for radiation-induced skin injury repair. Adv Healthc Mater 2023;12:e2203387. 36934301 10.1002/adhm.202203387

[13] Huang D , DuggalI, MatthewsBC, LeeE, TongE, PeppasNA. Rational design of stimuli responsive nanoparticle systems for the controlled, intracellular delivery of immunotherapeutic and chemotherapeutic agents. J Control Release 2025;384:113878.40441492 10.1016/j.jconrel.2025.113878

[14] Spałek M. Chronic radiation-induced dermatitis: challenges and solutions. Clin Cosmet Investig Dermatol 2016;9:473–82. 10.2147/CCID.S94320 PMC5161339 28003769

[15] Li X , Zhang X, Qi Y, Jin W, Wen Z, Zhao Y, Li X, Yao X, Shen Z, Zhang F, Lu P, Huang N, Wang X, Liu Y. Conductive bioadhesive hydrogel with controlled astragaloside IV release for ferroptosis-mediated cardiac repair. J Control Release 2025;384:113874. 40414503 10.1016/j.jconrel.2025.113874

[16] Olteanu G , Neacșu SM, Joița FA, Musuc AM, Lupu EC, Ioniță-Mîndrican C-B, Lupuliasa D, Mititelu M. Advancements in regenerative hydrogels in skin wound treatment: a comprehensive review. Int J Mol Sci 2024;25:3849. 38612660 10.3390/ijms25073849 PMC11012090

[17] Huang H , Qi X, Chen Y, Wu Z. Thermo-sensitive hydrogels for delivering biotherapeutic molecules: a review. Saudi Pharm J 2019;27:990–9. 31997906 10.1016/j.jsps.2019.08.001 PMC6978621

[18] Xu M , Fu T, Zhang C, An Z, Yan J, Lu Z, Wu H, Liu J, Qiu L, Shi L, Lin J, Cao Y, Pei R. Prolonged, staged, and self-regulated methotrexate release coupled with ROS scavenging in an injectable hydrogel for rheumatoid arthritis therapy. J Control Release 2024;375:60–73. 39216600 10.1016/j.jconrel.2024.08.046

[19] Kim JM , Kim SA, Kwon HJ, Moon S-H, Oh DY, Rhie JW, Jun Y-J. Reconstruction of radiation-induced ulcers with free flaps using the perforating vessel as a recipient vessel. Microsurgery 2019;39:613–20. 31441097 10.1002/micr.30504

[20] Yan T , Yang P, Bai H, Song B, Liu Y, Wang J, Zhang Y, Tu W, Yu D, Zhang S. Single-cell RNA-seq analysis of molecular changes during radiation-induced skin injury: the involvement of Nur77. Theranostics 2024;14:5809–25. 39346541 10.7150/thno.100417 PMC11426238

[21] Xu Y , Liu Q, Li W, Hu Z, Shi C. Recent advances in the mechanisms, current treatment status, and application of multifunctional biomaterials for radiation-induced skin injury. Theranostics 2025;15:2700–19. 40083928 10.7150/thno.108309 PMC11898283

[22] Shang J , Jin C, Wang F, Zhang Z, Xie L, Zhou Q, Wang Y, Xu X, Zhang S, Li J. Exploring the influence of growth factors in diabetic foot: a comprehensive bibliometric analysis. Medicine (Baltimore) 2025;104:e42716. 40760573 10.1097/MD.0000000000042716 PMC12323937

[23] Akkus G , Sert M. Diabetic foot ulcers: a devastating complication of diabetes mellitus continues non-stop in spite of new medical treatment modalities. World J Diabetes 2022;13:1106–21. 36578865 10.4239/wjd.v13.i12.1106 PMC9791571

[24] Kameni LE , Januszyk M, Berry CE, Downer MA, Parker JB, Morgan AG, Valencia C, Griffin M, Li DJ, Liang NE, Momeni A, Longaker MT, Wan DC. A review of radiation-induced vascular injury and clinical impact. Ann Plast Surg 2024;92:181–5. 37962260 10.1097/SAP.0000000000003723

[25] Ramia P , Bodgi L, Mahmoud D, Mohammad MA, Youssef B, Kopek N, Al-Shamsi H, Dagher M, Abu-Gheida I. Radiation-induced fibrosis in patients with head and neck cancer: a review of pathogenesis and clinical outcomes. Clin Med Insights Oncol 2022;16:11795549211036898. 35125900 10.1177/11795549211036898 PMC8808018

[26] Cui J , Wang T-J, Zhang Y-X, She L-Z, Zhao Y-C. Molecular biological mechanisms of radiotherapy-induced skin injury occurrence and treatment. Biomed Pharmacother 2024;180:117470. 39321513 10.1016/j.biopha.2024.117470

[27] Wijerathne H , Langston JC, Yang Q, Sun S, Miyamoto C, Kilpatrick LE, Kiani MF. Mechanisms of radiation-induced endothelium damage: emerging models and technologies. Radiother Oncol 2021;158:21–32. 33581220 10.1016/j.radonc.2021.02.007 PMC8119342

[28] Paldor M , Levkovitch-Siany O, Eidelshtein D, Adar R, Enk CD, Marmary Y, Elgavish S, Nevo Y, Benyamini H, Plaschkes I, Klein S, Mali A, Rose-John S, Peled A, Galun E, Axelrod JH. Single-cell transcriptomics reveals a senescence-associated IL-6/CCR6 axis driving radiodermatitis. EMBO Mol Med 2022;14:e15653. 35785521 10.15252/emmm.202115653 PMC9358397

[29] Liu G , Chen Y, Dai S, Wu G, Wang F, Chen W, Wu L, Luo P, Shi C. Targeting the NLRP3 in macrophages contributes to senescence cell clearance in radiation-induced skin injury. J Transl Med 2025;23:196. 39966955 10.1186/s12967-025-06204-z PMC11834210

[30] Borrelli MR , Shen AH, Lee GK, Momeni A, Longaker MT, Wan DC. Radiation-induced skin fibrosis: pathogenesis, current treatment options, and emerging therapeutics. Ann Plast Surg 2019;83:S59–64. 31513068 10.1097/SAP.0000000000002098 PMC6746243

[31] Jones EM , Cochrane CA, Percival SL. The effect of pH on the extracellular matrix and biofilms. Adv Wound Care (New Rochelle) 2015;4:431–9. 26155386 10.1089/wound.2014.0538 PMC4486717

[32] Guex AG , Poxson DJ, Simon DT, Berggren M, Fortunato G, Rossi RM, Maniura-Weber K, Rottmar M. Controlling pH by electronic ion pumps to fight fibrosis. Appl Mater Today 2021;22:100936.

[33] Dettmering T , Zahnreich S, Colindres-Rojas M, Durante M, Taucher-Scholz G, Fournier C. Increased effectiveness of carbon ions in the production of reactive oxygen species in normal human fibroblasts. J Radiat Res 2015;56:67–76. 25304329 10.1093/jrr/rru083 PMC4572590

[34] Liu J , Han X, Zhang T, Tian K, Li Z, Luo F. Reactive oxygen species (ROS) scavenging biomaterials for anti-inflammatory diseases: from mechanism to therapy. J Hematol Oncol 2023;16:116. 38037103 10.1186/s13045-023-01512-7 PMC10687997

[35] Cui Q , Wang X, Zhang Y, Shen Y, Qian Y. Macrophage-derived MMP-9 and MMP-2 are closely related to the rupture of the fibrous capsule of hepatocellular carcinoma leading to tumor invasion. Biol Proced Online 2023;25:8. 36918768 10.1186/s12575-023-00196-0 PMC10012540

[36] Bowser R , An J, Schwartz K, Sucholeiki RL, Sucholeiki I. ALS patients exhibit altered levels of total and active MMP-9 and several other biomarkers in serum and CSF compared to healthy controls and other neurologic diseases. Int J Mol Sci 2025;26:8900. 41009467 10.3390/ijms26188900 PMC12469682

[37] Wright JL , Takita C, Reis IM, Zhao W, Lee E, Nelson OL, Hu JJ. Prospective evaluation of radiation-induced skin toxicity in a race/ethnically diverse breast cancer population. Cancer Med 2016;5:454–64. 26763411 10.1002/cam4.608 PMC4799959

[38] Auerswald S , Schreml S, Meier R, Blancke Soares A, Niyazi M, Marschner S, Belka C, Canis M, Haubner F. Wound monitoring of pH and oxygen in patients after radiation therapy. Radiat Oncol 2019;14:199. 31711506 10.1186/s13014-019-1413-y PMC6849199

[39] Tian X , Guo J, Gu C, Wang H, Wang D, Liao Y, Zhu S, Zhao M, Gu Z. Ergothioneine-sodium hyaluronate dressing: a promising approach for protecting against radiation-induced skin injury. ACS Appl Mater Interfaces 2024;16:29917–29. 38813785 10.1021/acsami.4c05416

[40] Wang D , Wu W, Callen E, Pavani R, Zolnerowich N, Kodali S, Zong D, Wong N, Noriega S, Nathan WJ, Matos-Rodrigues G, Chari R, Kruhlak MJ, Livak F, Ward M, Caldecott K, Di Stefano B, Nussenzweig A. Active DNA demethylation promotes cell fate specification and the DNA damage response. Science 2022;378:983–9. 36454826 10.1126/science.add9838 PMC10196940

[41] Ameixa J , Bald I. Unraveling the complexity of DNA radiation damage using DNA nanotechnology. Acc Chem Res 2024;57:1608–19. 38780304 10.1021/acs.accounts.4c00121 PMC11154965

[42] Cao Z , Wang X, Jiang C, Wang H, Mu Y, Sun X, Chen X, Feng C. Thermo-sensitive hydroxybutyl chitosan/diatom biosilica hydrogel with immune microenvironment regulatory for chronic wound healing. Int J Biol Macromol 2024;262:130189. 38360227 10.1016/j.ijbiomac.2024.130189

[43] Rübe CE , Freyter BM, Tewary G, Roemer K, Hecht M, Rübe C. Radiation dermatitis: radiation-induced effects on the structural and immunological barrier function of the epidermis. Int J Mol Sci 2024;25:3320. 38542294 10.3390/ijms25063320 PMC10970573

[44] Habtezion A. Inflammation in acute and chronic pancreatitis. Curr Opin Gastroenterol 2015;31:395–9. 26107390 10.1097/MOG.0000000000000195 PMC4618697

[45] Zhou W , Zhu L, Zu X, Ma Y, Shen P, Hu Y, Zhang P, Ni Z, Wang N, Sun D, Bai Z, Gao X, Huangfu C, Gao Y. A novel antioxidant and anti-inflammatory carboxymethylcellulose/chitosan hydrogel loaded with cannabidiol promotes the healing of radiation-combined wound skin injury in the 60Co γ-irradiated mice. Phytomedicine 2025;142:156790. 40318533 10.1016/j.phymed.2025.156790

[46] Lai C-M , Chen W-J, Qin Y, Di Xu, Lai Y-K, He S-H. Innovative hydrogel design: tailoring immunomodulation for optimal chronic wound recovery. Adv Sci (Weinh) 2025;12:e2412360. 39575827 10.1002/advs.202412360 PMC11727140

[47] Jaberi A , Ghelich P, Samandari M, Kheirabadi S, Ataie Z, Kedzierski A, Hassani Najafabadi A, Tamayol A, Sheikhi A. Gelatin methacryloyl granular hydrogel scaffolds for skin wound healing. Biomater Sci 2025;13:6013–23. 40298015 10.1039/d4bm01062k PMC12038805

[48] Yu L , Dai Z, Huang Y, Tang S, Zhou L, Zhao X, Que X, Shi R, Zhou J, Dong J, Wang F, Gu Y. A temperature-sensitive chitosan hydrogels loaded with nano-zinc oxide and exosomes from human umbilical vein endothelial cells accelerates wound healing. Regen Ther 2025;30:63–74. 40491560 10.1016/j.reth.2025.04.020 PMC12146491

[49] Wang Y , Chen S, Bao S, Yao L, Wen Z, Xu L, Chen X, Guo S, Pang H, Zhou Y, Zhou P. Deciphering the fibrotic process: mechanism of chronic radiation skin injury fibrosis. Front Immunol 2024;15:1338922. 38426100 10.3389/fimmu.2024.1338922 PMC10902513

[50] Kim J-H , Nam J-K, Kim A-R, Park M-S, Lee H-J, Park J, Kim J, Lee Y-J. 2-methoxyestradiol inhibits radiation-induced skin injuries. Int J Mol Sci 2022;23:4171. 35456989 10.3390/ijms23084171 PMC9032705

[51] Farhood B , Ashrafizadeh M, Khodamoradi E, Hoseini-Ghahfarokhi M, Afrashi S, Musa AE, Najafi M. Targeting of cellular redox metabolism for mitigation of radiation injury. Life Sci 2020;250:117570. 32205088 10.1016/j.lfs.2020.117570

[52] Feng Z , Zhang Y, Yang C, Liu X, Huangfu Y, Zhang C, Huang P, Dong A, Liu J, Liu J, Kong D, Wang W. Bioinspired and inflammation-modulatory glycopeptide hydrogels for radiation-induced chronic skin injury repair. Adv Healthc Mater 2023;12:e2201671. 36183357 10.1002/adhm.202201671

[53] Vats K , Kruglov O, Mizes A, Samovich SN, Amoscato AA, Tyurin VA, Tyurina YY, Kagan VE, Bunimovich YL. Keratinocyte death by ferroptosis initiates skin inflammation after UVB exposure. Redox Biol 2021;47:102143. 34592565 10.1016/j.redox.2021.102143 PMC8487085

[54] Moon K-C , Yang J-P, Lee J-S, Jeong S-H, Dhong E-S, Han S-K. Effects of ultraviolet irradiation on cellular senescence in keratinocytes versus fibroblasts. J Craniofac Surg 2019;30:270–5. 30444781 10.1097/SCS.0000000000004904

[55] Lei G , Mao C, Yan Y, Zhuang L, Gan B. Ferroptosis, radiotherapy, and combination therapeutic strategies. Protein Cell 2021;12:836–57. 33891303 10.1007/s13238-021-00841-y PMC8563889

[56] Wei K , Nguyen HN, Brenner MB. Fibroblast pathology in inflammatory diseases. J Clin Invest 2021;131:e149538. 34651581 10.1172/JCI149538 PMC8516469

[57] Shaw R , Patel K, Chimthanawala NMA, Sathaye S, Maji SK. Peptide-based functional amyloid hydrogel enhances wound healing in normal and diabetic rat models. Adv Healthc Mater 2025;14:e2403560. 39935087 10.1002/adhm.202403560

[58] Koerdt S , Rohleder NH, Rommel N, Nobis C, Stoeckelhuber M, Pigorsch S, Duma M-N, Wolff K-D, Kesting MR. An expression analysis of markers of radiation-induced skin fibrosis and angiogenesis in wound healing disorders of the head and neck. Radiat Oncol 2015;10:202. 26390925 10.1186/s13014-015-0508-3 PMC4578371

[59] Chen Y. Hydrogels based on natural polymers for medicinal applications. Curr Med Chem 2020;27:2608–9. 32515308 10.2174/092986732716200604094035

[60] Wang J , Mao J, Du J, Zhao X. Review of gelatin hydrogel dressings based on cross-linking technology in the healing of hemorrhagic wounds: mechanism exploration, application research, and future perspectives. Macromol Rapid Commun 2025;46:e00326. 40518552 10.1002/marc.202500326

[61] Zhang M , Zhao X. Alginate hydrogel dressings for advanced wound management. Int J Biol Macromol 2020;162:1414–28. 32777428 10.1016/j.ijbiomac.2020.07.311

[62] Yuan N , Shao K, Huang S, Chen C. Chitosan, alginate, hyaluronic acid and other novel multifunctional hydrogel dressings for wound healing: a review. Int J Biol Macromol 2023;240:124321. 37019198 10.1016/j.ijbiomac.2023.124321

[63] Arabpour Z , Abedi F, Salehi M, Baharnoori SM, Soleimani M, Djalilian AR. Hydrogel-based skin regeneration. Int J Mol Sci 2024;25:1982. 38396661 10.3390/ijms25041982 PMC10888449

[64] Ahmed MS , Islam M, Hasan MK, Nam K-W. A comprehensive review of radiation-induced hydrogels: synthesis, properties, and multidimensional applications. Gels 2024;10:381. 38920928 10.3390/gels10060381 PMC11203285

[65] Pinnix C , Perkins GH, Strom EA, Tereffe W, Woodward W, Oh JL, Arriaga L, Munsell MF, Kelly P, Hoffman KE, Smith BD, Buchholz TA, Yu TK. Topical hyaluronic acid vs. standard of care for the prevention of radiation dermatitis after adjuvant radiotherapy for breast cancer: single-blind randomized phase III clinical trial. Int J Radiat Oncol Biol Phys 2012;83:1089–94. 22172912 10.1016/j.ijrobp.2011.09.021 PMC3935608

[66] Maeso L , Antezana PE, Hvozda Arana AG, Evelson PA, Orive G, Desimone MF. Progress in the use of hydrogels for antioxidant delivery in skin wounds. Pharmaceutics 2024;16:524. 38675185 10.3390/pharmaceutics16040524 PMC11053627

[67] Yan X , Guo Y-X, Liu Y-X, Liu C. Mesenchymal stem cell-derived exosomes and the wnt/β-catenin pathway: unifying mechanisms of multi-organ regeneration and the path to precision clinical translation. World J Stem Cells 2025;17:106902. 40585951 10.4252/wjsc.v17.i6.106902 PMC12203135

[68] Mamun AA , Shao C, Geng P, Wang S, Xiao J. Recent advances in molecular mechanisms of skin wound healing and its treatments. Front Immunol 2024;15:1395479. 38835782 10.3389/fimmu.2024.1395479 PMC11148235

[69] Fan Y , Han Q, Li H, Cai X, Dyett B, Qiao R, Drummond CJ, Thang SH, Zhai J. Recent developments in nanoparticle-hydrogel hybrid materials for controlled release. Adv Sci (Weinh) 2025;12:e07209. 40734635 10.1002/advs.202507209 PMC12463025

[70] Yan L , Wang J, Cai X, Liou Y-C, Shen H-M, Hao J, Huang C, Luo G, He W. Macrophage plasticity: signaling pathways, tissue repair, and regeneration. MedComm (2020) 2024;5:e658. 39092292 10.1002/mco2.658 PMC11292402

[71] Xia Y , Gui H, Li X, Wu Y, Liu J, Liu J. X-ray responsive antioxidant drug-free hydrogel for treatment of radiation skin injury. ACS Appl Mater Interfaces 2025;17:5671–83. 39825803 10.1021/acsami.4c16810

[72] Xue L , An R, Zhao J, Qiu M, Wang Z, Ren H, Yu D, Zhu X. Self-healing hydrogels: mechanisms and biomedical applications. MedComm (2020) 2025;6:e70181. 40276645 10.1002/mco2.70181 PMC12018771

[73] Alberts A , Tudorache D-I, Niculescu A-G, Grumezescu AM. Advancements in wound dressing materials: highlighting recent progress in hydrogels, foams, and antimicrobial dressings. Gels 2025;11:123. 39996666 10.3390/gels11020123 PMC11854827

[74] Dell AC , Wagner G, Own J, Geibel JP. 3D bioprinting using hydrogels: cell inks and tissue engineering applications. Pharmaceutics 2022;14:2596. 36559090 10.3390/pharmaceutics14122596 PMC9784738

[75] Souri M , Soltani M, Moradi Kashkooli F, Kiani Shahvandi M, Chiani M, Shariati FS, Mehrabi MR, Munn LL. Towards principled design of cancer nanomedicine to accelerate clinical translation. Mater Today Bio 2022;13:100208. 10.1016/j.mtbio.2022.100208 PMC8841842 35198957

[76] Narayanan KB , Bhaskar R. Innovations in designing hydrogels for advanced wound dressing applications: an editorial review. Gels 2025;11:332. 40422351 10.3390/gels11050332 PMC12111174

[77] Negut I , Dorcioman G, Grumezescu V. Scaffolds for wound healing applications. Polymers (Basel) 2020;12:2010. 32899245 10.3390/polym12092010 PMC7563417

[78] Grip J , Engstad RE, Skjæveland I, Škalko-Basnet N, Holsæter AM. Sprayable carbopol hydrogel with soluble beta-1,3/1,6-glucan as an active ingredient for wound healing - development and in-vivo evaluation. Eur J Pharm Sci 2017;107:24–31. 28645493 10.1016/j.ejps.2017.06.029

[79] He X , Hu X, Dong X, Li S, Ni Y. Application and clinical efficacy of sufu medical chitosan hydrogel dressing in radiation skin damage caused by radiotherapy for cervical cancer. Pak J Med Sci 2022;38:2331–6. 36415280 10.12669/pjms.38.8.5569 PMC9676621

[80] Ye J , Li Q, Zhang Y, Su Q, Feng Z, Huang P, Zhang C, Zhai Y, Wang W. ROS scavenging and immunoregulative EGCG@cerium complex loaded in antibacterial polyethylene glycol-chitosan hydrogel dressing for skin wound healing. Acta Biomater 2023;166:155–66. 37230435 10.1016/j.actbio.2023.05.027

[81] Zuo J , Du L, Li M, Liu B, Zhu W, Jin Y. Transdermal enhancement effect and mechanism of iontophoresis for non-steroidal anti-inflammatory drugs. Int J Pharm 2014;466:76–82. 24607207 10.1016/j.ijpharm.2014.03.013

[82] Qiao B , Wang J, Qiao L, Maleki A, Liang Y, Guo B. ROS-responsive hydrogels with spatiotemporally sequential delivery of antibacterial and anti-inflammatory drugs for the repair of MRSA-infected wounds. Regen Biomater 2024;11:rbad110. 38173767 10.1093/rb/rbad110 PMC10761208

[83] Guo J , Zhang X, Mao R, Li H, Hao Y, Zhang J, Wang W, Zhang Y, Liu J. Multifunctional glycopeptide-based hydrogel via dual-modulation for the prevention and repair of radiation-induced skin injury. ACS Biomater Sci Eng 2024;10:5168–80. 39016069 10.1021/acsbiomaterials.4c00698

[84] Jiao S , Li C, Guo F, Zhang J, Zhang H, Cao Z, Wang W, Bu W, Lin M, Lü J, Zhou Z. SUN1/2 controls macrophage polarization via modulating nuclear size and stiffness. Nat Commun 2023;14:6416. 37828059 10.1038/s41467-023-42187-5 PMC10570371

[85] Yu D , Li S, Wang S, Li X, Zhu M, Huang S, Sun L, Zhang Y, Liu Y, Wang S. Development and characterization of VEGF165-chitosan nanoparticles for the treatment of radiation-induced skin injury in rats. Mar Drugs 2016;14:182. 27727163 10.3390/md14100182 PMC5082330

[86] Tang A , Li Y, Yao Y, Yang X, Cao Z, Nie H, Yang G. Injectable keratin hydrogels as hemostatic and wound dressing materials. Biomater Sci 2021;9:4169–77. 33977985 10.1039/d1bm00135c

[87] Pazdrowski J , Gornowicz-Porowska J, Kaźmierska J, Krajka-Kuźniak V, Polanska A, Masternak M, Szewczyk M, Golusiński W, Danczak-Pazdrowska A. Radiation-induced skin injury in the head and neck region: pathogenesis, clinics, prevention, treatment considerations and proposal for management algorithm. Rep Pract Oncol Radiother 2024;29:373–90. 39144266 10.5603/rpor.100775 PMC11321788

[88] Fan R , Zhang C, Li F, Li B, McCarthy A, Zhang Y, Chen S, Zhang L. Hierarchically assembled nanofiber scaffolds with dual growth factor gradients promote skin wound healing through rapid cell recruitment. Adv Sci (Weinh) 2024;11:e2309993. 38326085 10.1002/advs.202309993 PMC11005683

[89] Wang H , Zhang N, Wang X, Tian J, Yi J, Yao L, Huang G. Emerging role of mesenchymal stem cell-derived exosome microRNA in radiation injury. Int J Radiat Biol 2024;100:996–1008. 38776447 10.1080/09553002.2024.2347348

[90] Liao F , Chen L, Luo P, Jiang Z, Chen Z, Wang Z, Zhang C, Wang Y, He J, Wang Q, Wang Y, Liu L, Huang Y, Wang H, Jiang Q, Luo M, Gan Y, Liu Y, Wang Y, Wu J, Xie W, Cheng Z, Dai Y, Li J, Liu Z, Yang F, Shi C. PC4 serves as a negative regulator of skin wound healing in mice. Burns Trauma 2020;8:tkaa010. 32373645 10.1093/burnst/tkaa010 PMC7198317

[91] Jimenez-Socha M , Dion GR, Mora-Navarro C, Wang Z, Nolan MW, Freytes DO. Radiation-induced fibrosis in head and neck cancer: challenges and future therapeutic strategies for vocal fold treatments. Cancers (Basel) 2025;17:1108. 40227628 10.3390/cancers17071108 PMC11987993

[92] Che X , Zhao T, Hu J, Yang K, Ma N, Li A, Sun Q, Ding C, Ding Q. Application of chitosan-based hydrogel in promoting wound healing: a review. Polymers (Basel) 2024;16:344. 38337233 10.3390/polym16030344 PMC10857268

[93] DeCostanza L , Grogan GM, Bruce AC, Peachey CM, Clark EA, Atkins K, Tylek T, Solga MD, Spiller KL, Peirce SM, Campbell CA, Cottler PS. Decellularized porcine dermal hydrogel enhances implant-based wound healing in the setting of irradiation. Acta Biomater 2025;191:260–75. 39522628 10.1016/j.actbio.2024.11.009 PMC11659024

[94] Stefanov I , Pérez-Rafael S, Hoyo J, Cailloux J, Santana Pérez OO, Hinojosa-Caballero D, Tzanov T. Multifunctional enzymatically generated hydrogels for chronic wound application. Biomacromolecules 2017;18:1544–55. 28421746 10.1021/acs.biomac.7b00111

[95] Choi H , Choi W-S, Jeong J-O. A review of advanced hydrogel applications for tissue engineering and drug delivery systems as biomaterials. Gels 2024;10:693. 39590049 10.3390/gels10110693 PMC11594258

[96] Geng H , Zheng X, Zhang Y, Cui X, Li Z, Zhang X, Cui J, Meng F, Sun L, Ni S. Microenvironment‐responsive hydrogels with detachable skin adhesion and mild-temperature photothermal property for chronic wound healing. Adv Funct Mater 2023;33:17.

[97] Amanzholkyzy A , Zhumagaliyeva S, Sultanova N, Abilov Z, Ongalbek D, Donbayeva E, Niyazbekova A, Mukazhanova Z. Hydrogel delivery systems for biological active substances: properties and the role of HPMC as a carrier. Molecules 2025;30:1354. 40142128 10.3390/molecules30061354 PMC11946135

[98] Zhou D , Li Z, Bao L, Zhao X, Hao J, Xu C, Sun F, He D, Jiang C, Zeng T, Li D. DNAzyme hydrogels specifically inhibit the NLRP3 pathway to prevent radiation-induced skin injury in mice. J Nanobiotechnology 2025;23:238. 40119386 10.1186/s12951-025-03147-x PMC11929335

[99] Shi Y , Tang Z, Li C, Miao Y, Zhu L, Yue B. Conductive hydrogel systems enabling rapid and controllable release of guests at low-voltage region. Small 2025;21:e2410120. 39778018 10.1002/smll.202410120

[100] Liang C , Dudko V, Khoruzhenko O, Hong X, Lv Z-P, Tunn I, Umer M, Timonen JVI, Linder MB, Breu J, Ikkala O, Zhang H. Stiff and self-healing hydrogels by polymer entanglements in co-planar nanoconfinement. Nat Mater 2025;24:599–606. 40055539 10.1038/s41563-025-02146-5 PMC11961364

[101] Yuan Y , Shi Y, Banerjee J, Sadeghpour A, Azevedo HS. Structuring supramolecular hyaluronan hydrogels via peptide self-assembly for modulating the cell microenvironment. Mater Today Bio 2023;19:100598. 10.1016/j.mtbio.2023.100598 PMC10024175 36942310

[102] Setoyama S , Haraguchi R, Aoki S, Oishi Y, Narita T. pH-responsive collagen hydrogels prepared by UV irradiation in the presence of riboflavin. Int J Mol Sci 2024;25:10439. 39408768 10.3390/ijms251910439 PMC11476811

[103] Yang C , Saiding Q, Chen W, An S, Zhao S, Khan MM, Kong N, Ge M, Shi J, Lin H, Tao W. Chemically modified and inactivated bacteria enable intra-biofilm drug delivery and long-term immunity against implant infections. Nat Biomed Eng 2026 Jan 16. [Epub ahead of print]. 10.1038/s41551-025-01600-8 41545779

[104] Jia X , Dou Z, Zhang Y, Li F, Xing B, Hu Z, Li X, Liu Z, Yang W, Liu Z. Smart responsive and controlled-release hydrogels for chronic wound treatment. Pharmaceutics 2023;15:2735. 38140076 10.3390/pharmaceutics15122735 PMC10747460

[105] Cui X , Wang J, Xu X, Cao X, Zhou Y, Guo J. Progress and application of multifunctional hydrogel in radioactive skin injury. Adv Mater Inter 2025;12:2400976.

[106] Shi Y , Lin R, Cui H, Azevedo HS. Multifunctional self-assembling peptide-based nanostructures for targeted intracellular delivery: design, physicochemical characterization, and biological assessment. Methods Mol Biol 2018;1758:11–26. 29679319 10.1007/978-1-4939-7741-3_2

[107] Ren H , Zhang Z, Chen X, He C. Stimuli-responsive hydrogel adhesives for wound closure and tissue regeneration. Macromol Biosci 2024;24:e2300379. 37827713 10.1002/mabi.202300379 PMC13420974

[108] Khattak S , Ullah I, Sohail M, Akbar MU, Rauf MA, Ullah S, Shen J, Xu H-T. Endogenous/exogenous stimuli-responsive smart hydrogels for diabetic wound healing. Aggregate 2025;6:e688.

[109] Wang J , Lin Y, Fan H, Cui J, Wang Y, Wang Z. ROS/pH dual-responsive hydrogel dressings loaded with amphiphilic structured nano micelles for the repair of infected wounds. Int J Nanomedicine 2025;20:8119–42. 40584783 10.2147/IJN.S522589 PMC12205371

[110] Ruan Z , Shi T, Guo Z, Zhu Y, Wang W, Ma Y, Ding C, Zhang Y, Wang X, Chen Y, Lin H, Ge M. Oxygen-immunomodulated nanocatalysts enhance anti-infection by trained immunity. Adv Funct Mater 2025;35:e09454.

[111] Ge M , Guo H, Zong M, Chen Z, Liu Z, Lin H, Shi J. Bandgap-engineered germanene nanosheets as an efficient photodynamic agent for cancer therapy. Angew Chem Int Ed 2023;62:e202215795. 10.1002/anie.202215795 36624080

[112] Song W , Zhang C, Li Z, Li K, Kong Y, Du J, Kong Y, Guo X, Ju X, Zhu M, Tian Y, Huang S, Niu Z. pH-responsive hydrogel with dual-crosslinked network of polyvinyl alcohol/boric acid for controlled release of salvianolic acid B: novel pro-regenerative mechanisms in scar inhibition and wound healing. Regen Biomater 2025;12:rbaf002. 39897539 10.1093/rb/rbaf002 PMC11785367

[113] Gao K , Xu K. Advancements and prospects of pH-responsive hydrogels in biomedicine. Gels 2025;11:293. 40277729 10.3390/gels11040293 PMC12026617

[114] Ding K , Liao M, Wang Y, Lu JR. Advances in composite stimuli-responsive hydrogels for wound healing: mechanisms and applications. Gels 2025;11:11. 10.3390/gels11060420 PMC12191909 40558719

[115] Fan Y , Wang H, Wang C, Xing Y, Liu S, Feng L, Zhang X, Chen J. Advances in smart-response hydrogels for skin wound repair. Polymers (Basel) 2024;16:2818. 39408528 10.3390/polym16192818 PMC11479249

[116] Du N , Fan Y, Huang H, Guan Y, Nan K. Stimuli-responsive hydrogel actuators for skin therapeutics and beyond. Soft Sci 2024;4:35.

[117] Zhao Y , Tan Y, Zeng C, Pan W. Ultrafast enzyme-responsive hydrogel for real-time assessment and treatment optimization in infected wounds. J Nanobiotechnology 2025;23:9. 39780182 10.1186/s12951-024-03078-z PMC11716278

[118] Jahanbekam S , Asare-Addo K, Alipour S, Nokhodchi A. Smart hydrogels and the promise of multi-responsive in-situ systems. J Drug Deliv Sci Technol 2025;107:106758.

[119] Luo X , Liu H, Wen J, Hu J, Li Y, Li G, Dai G, Li Y, Li J. Composite hydrogels with antioxidant and robust adhesive properties for the prevention of radiation-induced dermatitis. J Mater Chem B 2024;12:6927–39. 38904166 10.1039/d4tb00511b

[120] Ge M , Zhu W, Mei J, Hu T, Yang C, Lin H, Shi J. Piezoelectric-enhanced nanocatalysts trigger neutrophil N1 polarization against bacterial biofilm by disrupting redox homeostasis. Adv Mater 2025;37:e2409633. 39350533 10.1002/adma.202409633

[121] Protsak IS , Morozov YM. Fundamentals and advances in stimuli-responsive hydrogels and their applications: a review. Gels 2025;11:30. 39852001 10.3390/gels11010030 PMC11765116

[122] Wang S , Wu W-Y, Yeo JCC, Soo XYD, Thitsartarn W, Liu S, Tan BH, Suwardi A, Li Z, Zhu Q, Loh XJ. Responsive hydrogel dressings for intelligent wound management. BMEMat 2023;1:e12021.

[123] Yang S , Sun Y, Yan C. Recent advances in the use of extracellular vesicles from adipose-derived stem cells for regenerative medical therapeutics. J Nanobiotechnology 2024;22:316. 38844939 10.1186/s12951-024-02603-4 PMC11157933

[124] Li Z , Liu J, Song J, Yin Z, Zhou F, Shen H, Wang G, Su J. Multifunctional hydrogel-based engineered extracellular vesicles delivery for complicated wound healing. Theranostics 2024;14:4198–217. 39113809 10.7150/thno.97317 PMC11303081

[125] Pan J , Han X, Yuan P, Xu Y, Wang W, Zhang Z, Yang G, Huang L, Yang Y. A multifunctional hydrogel microneedle patch for treatment of atopic dermatitis. ACS Appl Mater Interfaces 2025;17:29341–51. 40327888 10.1021/acsami.5c03594

[126] Shi Y , Zheng Z, Li Y, Wang Y, Azevedo HS, Liu X, Zhang C, Hu H. Bacterial extracellular vesicles as bioactive nanocarriers for wound treatment. Acta Pharm Sin B 2026 January 12.[Epub ahead of print] 10.1016/j.apsb.2026.01.007 PMC13031071 41909759

[127] Tang J , Jia X, Li Q, Cui Z, Liang A, Ke B, Yang D, Yao C. A DNA-based hydrogel for exosome separation and biomedical applications. Proc Natl Acad Sci USA 2023;120:e2303822120. 37399419 10.1073/pnas.2303822120 PMC10334772

[128] Ryan P , Lee J. In vitro senescence and senolytic functional assays. Biomater Sci 2025;13:3509–31. 40375674 10.1039/d4bm01684j PMC12197831

[129] Zhang L , Pitcher LE, Prahalad V, Niedernhofer LJ, Robbins PD. Targeting cellular senescence with senotherapeutics: senolytics and senomorphics. FEBS J 2023;290:1362–83. 35015337 10.1111/febs.16350

[130] Garau Paganella L , Bovone G, Cuni F, Labouesse C, Cui Y, Giampietro C, Tibbitt MW. Injectable senolytic hydrogel depot for the clearance of senescent cells. Biomacromolecules 2025;26:814–24. 39783796 10.1021/acs.biomac.4c00851 PMC11815846

[131] Chen Z , Wang L, Guo C, Qiu M, Cheng L, Chen K, Qi J, Deng L, He C, Li X, Yan Y. Vascularized polypeptide hydrogel modulates macrophage polarization for wound healing. Acta Biomater 2023;155:218–34. 36396041 10.1016/j.actbio.2022.11.002

[132] Shen J , Jiao W, Yang J, Zhuang B, Du S, Wu Y, Huang G, Zhang Y, Wang Y, Xu C, Du L, Jin Y. In situ photocrosslinkable hydrogel treats radiation-induced skin injury by ROS elimination and inflammation regulation. Biomaterials 2025;314:122891. 39413652 10.1016/j.biomaterials.2024.122891

[133] Li Z , Zheng A, Liang C, Mao Z, Deng T, Cao L, Wang C. ROS scavenging Mn_3_O_4_ nanozyme regulated immune microenvironment and affects intercellular interaction to promote wound healing in diabetes. Regen Biomater 2025;12:rbaf089. 41048976 10.1093/rb/rbaf089 PMC12490823

[134] Hao J , Sun M, Li D, Zhang T, Li J, Zhou D. An IFI6-based hydrogel promotes the healing of radiation-induced skin injury through regulation of the HSF1 activity. J Nanobiotechnology 2022;20:288. 35717249 10.1186/s12951-022-01466-x PMC9206756

[135] Serpico L , Dello Iacono S, Cammarano A, Stefano LD. Recent advances in stimuli-responsive hydrogel-based wound dressing. Gels 2023;9:451. 37367122 10.3390/gels9060451 PMC10298082

[136] Huang C , Huangfu C, Bai Z, Zhu L, Shen P, Wang N, Li G, Deng H, Ma Z, Zhou W, Gao Y. Multifunctional carbomer based ferulic acid hydrogel promotes wound healing in radiation-induced skin injury by inactivating NLRP3 inflammasome. J Nanobiotechnology 2024;22:576. 39300534 10.1186/s12951-024-02789-7 PMC11411768

[137] Yao W-D , Zhou J-N, Tang C, Zhang J-L, Chen Z-Y, Li Y, Gong X-J, Qu M-Y, Zeng Q, Jia Y-L, Wang H-Y, Fan T, Ren J, Guo L-L, Xi J-F, Pei X-T, Han Y, Yue W. Hydrogel microneedle patches loaded with stem cell mitochondria-enriched microvesicles boost the chronic wound healing. ACS Nano 2024;18:26733–50. 39238258 10.1021/acsnano.4c06921 PMC11447894

[138] Kim S , Lee H-Y, Lee HR, Jang JY, Yun JH, Shin YS, Kim C-H. Liquid-type plasma-controlled in situ crosslinking of silk-alginate injectable gel displayed better bioactivities and mechanical properties. Mater Today Bio 2022;15:100321. 10.1016/j.mtbio.2022.100321 PMC9214807 35757030

[139] Ge M , Ruan Z, Zhu Y-X, Wu W, Yang C, Lin H, Shi J. A natural killer cell mimic against intracellular pathogen infections. Sci Adv 2024;10:eadp3976. 39475620 10.1126/sciadv.adp3976 PMC11524181

[140] Guo D , Daman K, Chen JJ, Shi M-J, Yan J, Matijasevic Z, Rickard AM, Bennett MH, Kiselyov A, Zhou H, Bang AG, Wagner KR, Maehr R, King OD, Hayward LJ, Emerson CP. iMyoblasts for ex vivo and in vivo investigations of human myogenesis and disease modeling. Elife 2022;11:e70341. 35076017 10.7554/eLife.70341 PMC8789283

[141] Liao H , Hu S, Yang H, Wang L, Tanaka S, Takigawa I, Li W, Fan H, Gong JP. Data-driven de novo design of super-adhesive hydrogels. Nature 2025;644:89–95. 40770436 10.1038/s41586-025-09269-4 PMC12328221

[142] Ausri IR , Sadeghzadeh S, Biswas S, Zheng H, GhavamiNejad P, Huynh MDT, Keyvani F, Shirzadi E, Rahman FA, Quadrilatero J, GhavamiNejad A, Poudineh M. Multifunctional dopamine-based hydrogel microneedle electrode for continuous ketone sensing. Adv Mater 2024;36:e2402009. 38847967 10.1002/adma.202402009

[143] Sumey JL , Johnston PC, Harrell AM, Caliari SR. Hydrogel mechanics regulate fibroblast DNA methylation and chromatin condensation. Biomater Sci 2023;11:2886–97. 36880435 10.1039/d2bm02058k PMC10329270

[144] Laggner M , Acosta GS, Kitzmüller C, Copic D, Gruber F, Altenburger LM, Vorstandlechner V, Gugerell A, Direder M, Klas K, Bormann D, Peterbauer A, Shibuya A, Bohle B, Ankersmit HJ, Mildner M. The secretome of irradiated peripheral blood mononuclear cells attenuates activation of mast cells and basophils. EBioMedicine 2022;81:104093. 35671621 10.1016/j.ebiom.2022.104093 PMC9168057

[145] Yu H , Zhong T, Xu Y, Zhang Z, Ma J, Yuan J, Wang M, Wu M, Yu J, Ma Y, Chen D. Molecular profiling of skin cells identifies distinct cellular signatures in radiation-induced skin injury across various stages in the murine dataset. Exp Hematol Oncol 2025;14:18. 40001256 10.1186/s40164-025-00596-w PMC11852861

[146] Shen B , Qian B, Tu N. Utilizing AI algorithms to model and optimize the composite of nanocellulose and hydrogels via a new technique. Int J Biol Macromol 2025;290:138903. 39701236 10.1016/j.ijbiomac.2024.138903

[147] Li Z , Song P, Li G, Han Y, Ren X, Bai L, Su J. AI energized hydrogel design, optimization and application in biomedicine. Mater Today Bio 2024;25:101014. 10.1016/j.mtbio.2024.101014 PMC10924066 38464497

[148] Shi Y , Hu H. AI accelerated discovery of self-assembling peptides. Biomater Transl 2023;4:291–3. 38282703 10.12336/biomatertransl.2023.04.008 PMC10817794

[149] Lin N , Abbas-Aghababazadeh F, Su J, Wu AJ, Lin C, Shi W, Xu W, Haibe-Kains B, Liu F-F, Kwan JYY. Development of machine learning models for predicting radiation dermatitis in breast cancer patients using clinical risk factors, patient-reported outcomes, and serum cytokine biomarkers. Clin Breast Cancer 2025;25:e622–34.e6. 40155248 10.1016/j.clbc.2025.03.002

[150] Lee T-F , Liu Y-H, Chang C-H, Chiu C-L, Lin C-H, Shao J-C, Yen Y-C, Lin G-Z, Yang J, Tseng C-D, Fang F-M, Chao P-J, Lee S-H. Development of a risk prediction model for radiation dermatitis following proton radiotherapy in head and neck cancer using ensemble machine learning. Radiat Oncol 2024;19:78. 38915112 10.1186/s13014-024-02470-1 PMC11194979

[151] Collins GS , Moons KGM, Dhiman P, Riley RD, Beam AL, Van Calster B, Ghassemi M, Liu X, Reitsma JB, van Smeden M, Boulesteix A-L, Camaradou JC, Celi LA, Denaxas S, Denniston AK, Glocker B, Golub RM, Harvey H, Heinze G, Hoffman MM, Kengne AP, Lam E, Lee N, Loder EW, Maier-Hein L, Mateen BA, McCradden MD, Oakden-Rayner L, Ordish J, Parnell R, Rose S, Singh K, Wynants L, Logullo P. TRIPOD+AI statement: updated guidance for reporting clinical prediction models that use regression or machine learning methods. BMJ 2024;385:e078378. 38626948 10.1136/bmj-2023-078378 PMC11019967

[152] Liu X , Cruz Rivera S, Moher D, Calvert MJ, Denniston AK, SPIRIT-AI and CONSORT-AI Working Group Reporting guidelines for clinical trial reports for interventions involving artificial intelligence: the CONSORT-AI extension. Nat Med 2020;26:1364–74. 32908283 10.1038/s41591-020-1034-x PMC7598943

[153] Cruz Rivera S , Liu X, Chan A-W, Denniston AK, Calvert MJ, SPIRIT-AI and CONSORT-AI Consensus Group Guidelines for clinical trial protocols for interventions involving artificial intelligence: the SPIRIT-AI extension. Nat Med 2020;26:1351–63. 32908284 10.1038/s41591-020-1037-7 PMC7598944

[154] Faes L , Liu X, Wagner SK, Fu DJ, Balaskas K, Sim DA, Bachmann LM, Keane PA, Denniston AK. A clinician’s guide to artificial intelligence: how to critically appraise machine learning studies. Transl Vis Sci Technol 2020;9:7. 10.1167/tvst.9.2.7 PMC7346877 32704413

[155] Camacho-Cardenosa M , Pulido-Escribano V, Estrella-Guisado G, Dorado G, Herrera-Martínez AD, Gálvez-Moreno MÁ, Casado-Díaz A. Bioprinted hydrogels as vehicles for the application of extracellular vesicles in regenerative medicine. Gels 2025;11:191. 40136896 10.3390/gels11030191 PMC11941778

[156] Zhou X , Yu X, You T, Zhao B, Dong L, Huang C, Zhou X, Xing M, Qian W, Luo G. 3D printing-based hydrogel dressings for wound healing. Adv Sci 2024;11:2404580. 10.1002/advs.202404580 PMC11653715 39552255

[157] Li W , Wang M, Ma H, Chapa-Villarreal FA, Lobo AO, Zhang YS. Stereolithography apparatus and digital light processing-based 3D bioprinting for tissue fabrication. iScience 2023;26:106039. 36761021 10.1016/j.isci.2023.106039 PMC9906021

[158] Lv X , Zhao N, Long S, Wang G, Ran X, Gao J, Wang J, Wang T. 3D skin bioprinting as promising therapeutic strategy for radiation-associated skin injuries. Wound Repair Regen 2024;32:217–28. 38602068 10.1111/wrr.13181

[159] Zhang T , Sheng S, Cai W, Yang H, Li J, Niu L, Chen W, Zhang X, Zhou Q, Gao C, Li Z, Zhang Y, Wang G, Shen H, Zhang H, Hu Y, Yin Z, Chen X, Liu Y, Cui J, Su J. 3-D bioprinted human-derived skin organoids accelerate full-thickness skin defects repair. Bioact Mater 2024;42:257–69. 39285913 10.1016/j.bioactmat.2024.08.036 PMC11404058

[160] Zhao M , Wang J, Zhang J, Huang J, Luo L, Yang Y, Shen K, Jiao T, Jia Y, Lian W, Li J, Wang Y, Lian Q, Hu D. Functionalizing multi-component bioink with platelet-rich plasma for customized in-situ bilayer bioprinting for wound healing. Mater Today Bio 2022;16:100334. 10.1016/j.mtbio.2022.100334 PMC9254123 35799896

[161] Hu F , Gao Q, Liu J, Chen W, Zheng C, Bai Q, Sun N, Zhang W, Zhang Y, Lu T. Smart microneedle patches for wound healing and management. J Mater Chem B 2023;11:2830–51. 36916631 10.1039/d2tb02596e

[162] Ma Z, , Chen Y, , Tang K, , Yang H, , Tian M, , Xi X, , Han S, , Yang S, , Ru L, , Yu X. Highly efficient prevention of radiation dermatitis using a pegylated superoxide dismutase dissolving microneedle patch. Eur J Pharm Biopharm 2024;201:114347. 38825168 10.1016/j.ejpb.2024.114347

[163] Mohite P , Puri A, Munde S, Ade N, Kumar A, Jantrawut P, Singh S, Chittasupho C. Hydrogel-forming microneedles in the management of dermal disorders through a non-invasive process: a review. Gels 2024;10:719. 39590075 10.3390/gels10110719 PMC11594199

[164] Cristóbal L , Asúnsolo Á, Sánchez J, Ortega MA, Álvarez-Mon M, García-Honduvilla N, Buján J, Maldonado AA. Mouse models for human skin transplantation: a systematic review. Cells Tissues Organs 2021;210:250–9. 34521089 10.1159/000516154

[165] Jiao T , Sun C, Wang Z, Han G, Wang H. 3D bioprinted alginate/gelatin hydrogel: concentration modulated properties toward scar-minimized wound healing. J Biomater Sci Polym Ed 2025;36:2137–58. 40238648 10.1080/09205063.2025.2491609

[166] Hemmati Dezaki Z , Parivar K, Goodarzi V, Nourani MR. Cobalt/bioglass nanoparticles enhanced dermal regeneration in a 3-layered electrospun scaffold. Adv Pharm Bull 2024;14:192–207. 38585469 10.34172/apb.2024.006 PMC10997931

[167] Khan MUA , Stojanović GM, Abdullah MFB, Dolatshahi-Pirouz A, Marei HE, Ashammakhi N, Hasan A. Fundamental properties of smart hydrogels for tissue engineering applications: a review. Int J Biol Macromol 2024;254:127882. 37951446 10.1016/j.ijbiomac.2023.127882

[168] Egorikhina MN , Timofeeva LB, Rubtsova YP, Farafontova EA, Linkova DD, Charykova IN, Ryabkov MG, Ezhevskaya AA, Levicheva EA, Aleynik DY. Study of the effectiveness of skin restoration using a biopolymer hydrogel scaffold with encapsulated mesenchymal stem cells. Int J Mol Sci 2025;26:7840. 40869164 10.3390/ijms26167840 PMC12386504

[169] Sparks HD , Sigaeva T, Tarraf S, Mandla S, Pope H, Hee O, Di Martino ES, Biernaskie J, Radisic M, Scott WM. Biomechanics of wound healing in an equine limb model: effect of location and treatment with a peptide-modified collagen-chitosan hydrogel. ACS Biomater Sci Eng 2021;7:265–78. 33342210 10.1021/acsbiomaterials.0c01431

[170] Murphy SV , Skardal A, Nelson RA, Sunnon K, Reid T, Clouse C, Kock ND, Jackson J, Soker S, Atala A. Amnion membrane hydrogel and amnion membrane powder accelerate wound healing in a full thickness porcine skin wound model. Stem Cells Transl Med 2020;9:80–92. 31328435 10.1002/sctm.19-0101 PMC6954699

[171] Riabinin A , Pankratova M, Rogovaya O, Vorotelyak E, Terskikh V, Vasiliev A. Ideal living skin equivalents, from old technologies and models to advanced ones: the prospects for an integrated approach. Biomed Res Int 2024;2024:9947692. 39184355 10.1155/2024/9947692 PMC11343635

[172] Hong Z-X , Zhu S-T, Li H, Luo J-Z, Yang Y, An Y, Wang X, Wang K. Bioengineered skin organoids: from development to applications. Mil Med Res 2023;10:40. 37605220 10.1186/s40779-023-00475-7 PMC10463602

[173] Quílez C , Jeon EY, Pappalardo A, Pathak P, Abaci HE. Efficient generation of skin organoids from pluripotent cells via defined extracellular matrix cues and morphogen gradients in a spindle-shaped microfluidic device. Adv Healthc Mater 2024;13:e2400405. 38452278 10.1002/adhm.202400405 PMC11305970

[174] Ruini C , Kendziora B, Ergun EZ, Sattler E, Gust C, French LE, Bağcı IS, Hartmann D. In vivo examination of healthy human skin after short-time treatment with moisturizers using confocal raman spectroscopy and optical coherence tomography: preliminary observations. Skin Res Technol 2022;28:119–32. 34555219 10.1111/srt.13101 PMC9907652

[175] Schmeel LC , Koch D, Stumpf S, Leitzen C, Simon B, Schüller H, Vornholt S, Schoroth F, Müdder T, Röhner F, Garbe S, Schmeel FC, Schild HH, Wilhelm-Buchstab TM. Prophylactically applied hydrofilm polyurethane film dressings reduce radiation dermatitis in adjuvant radiation therapy of breast cancer patients. Acta Oncol 2018;57:908–15. 29463159 10.1080/0284186X.2018.1441542

[176] Pazdrowski J , Polańska A, Kaźmierska J, Kowalczyk MJ, Szewczyk M, Niewinski P, Golusiński W, Dańczak-Pazdrowska A. The assessment of the long-term impact of radiotherapy on biophysical skin properties in patients after head and neck cancer. Medicina (Kaunas) 2024;60:739. 38792923 10.3390/medicina60050739 PMC11122895

[177] Macmillan MS , Wells M, MacBride S, Raab GM, Munro A, MacDougall H. Randomized comparison of dry dressings versus hydrogel in management of radiation-induced moist desquamation. Int J Radiat Oncol Biol Phys 2007;68:864–72. 17363185 10.1016/j.ijrobp.2006.12.049

[178] Tungkasamit T , Chakrabandhu S, Samakgarn V, Kunawongkrit N, Jirawatwarakul N, Chumachote A, Chitapanarux I. Reduction in severity of radiation-induced dermatitis in head and neck cancer patients treated with topical aloe vera gel: a randomized multicenter double-blind placebo-controlled trial. Eur J Oncol Nurs 2022;59:102164. 35767935 10.1016/j.ejon.2022.102164

[179] Menêses AG , Ferreira EB, Vieira LAC, Bontempo PdSM, Guerra ENS, Ciol MA, Reis PED. Comparison of liposomal gel with and without addition of chamomile for prevention of radiation dermatitis in head and neck cancer patients: a randomized controlled trial. Radiother Oncol 2024;199:110440. 39032836 10.1016/j.radonc.2024.110440

[180] Ahn S , Sung K, Kim HJ, Choi YE, Lee YK, Kim JS, Lee SK, Roh J-Y. Reducing radiation dermatitis using a film-forming silicone gel during breast radiotherapy: a pilot randomized-controlled trial. In Vivo 2020;34:413–22. 31882508 10.21873/invivo.11790 PMC6984096

[181] Herst P , van Schalkwyk M, Baker N, Thyne R, Dunne K, Moore K, Jackson F, Beaven K, Rutten L, McKee G, Willink R, James M. Mepitel film versus StrataXRT in managing radiation dermatitis in an intra-patient controlled clinical trial of 80 postmastectomy patients. J Med Imaging Radiat Oncol 2025;69:440–6. 40223795 10.1111/1754-9485.13850 PMC12175215

[182] Heydari B , Sheikhalishahi S, Hoseinzade F, Shabani M, Ramezani V, Saghafi F. Topical curcumin for prevention of radiation-induced dermatitis: a pilot double‑blind, placebo‑controlled trial. Cancer Invest 2025;43:173–82. 40145664 10.1080/07357907.2025.2479542

[183] Ren S , Jin J, Wu X, Han B, Zhang W, Rong F, Hou W, Shi Q, Lin H, Liu J. Effect of an herbal gel for the prevention of radiation dermatitis-related symptoms: an open-label randomized clinical trial. J Dermatolog Treat 2025;36:2489595. 40229671 10.1080/09546634.2025.2489595

[184] Li Q , Feng S, Yu Z, Wang Y, Tian L, Geng H, Guo C, Ning F, Luo J, Liu C. Amelioration of acute radiation dermatitis in breast cancer patients by a bioadhesive barrier-forming gel (episil): a single-center, open, parallel, randomized, phase I/II controlled trial. Clin Pharmacol Ther 2024;115:1085–91. 38159264 10.1002/cpt.3171

[185] Zöller K , To D, Bernkop-Schnürch A. Biomedical applications of functional hydrogels: innovative developments, relevant clinical trials and advanced products. Biomaterials 2025;312:122718. 39084097 10.1016/j.biomaterials.2024.122718

[186] Su Y , Cui H, Yang C, Li L, Xu F, Gao J, Zhang W. Hydrogels for the treatment of radiation-induced skin and mucosa damages: an up-to-date overview. Front Mater 2022;9:1018815.

[187] Sahin F , Pirouzpanah MB, Bijanpour H, Mohammadzadeh M, Eghdam Zamiri R, Ghasemi Jangjoo A, Nasiri B, Saboori H, Doğan A, Demirci S, Ayşan E, Çağrı Büke A, Naseri AR, Shakouri SK, Aghamohammadi D, Alizade-Harakiyan M, Seyed Nejad F. The preventive effects of boron-based gel on radiation dermatitis in patients being treated for breast cancer: a phase III randomized, double-blind, placebo-controlled clinical trial. Oncol Res Treat 2022;45:197–204. 34979503 10.1159/000520363

[188] Xiang Y , Zhang J, Mao H, Yan Z, Wang X, Bao C, Zhu L. Highly tough, stretchable, and enzymatically degradable hydrogels modulated by bioinspired hydrophobic β-sheet peptides. Biomacromolecules 2021;22:4846–56. 34706536 10.1021/acs.biomac.1c01134

[189] Stevens KR , Miller JS, Blakely BL, Chen CS, Bhatia SN. Degradable hydrogels derived from PEG-diacrylamide for hepatic tissue engineering. J Biomed Mater Res A 2015;103:3331–8. 25851120 10.1002/jbm.a.35478 PMC4890565

[190] Alonso JM , Andrade Del Olmo J, Perez Gonzalez R, Saez-Martinez V. Injectable hydrogels: from laboratory to industrialization. Polymers (Basel) 2021;13:650. 33671648 10.3390/polym13040650 PMC7926321

[191] Zheng B , Zhang P, Lv Q, Wu T, Liu Y, Tang J, Ma Y, Cheng L, Xu L, Wang Y, Xue Y, Liu J, Ren J. Development and preclinical evaluation of multifunctional hydrogel for precise thermal protection during thermal ablation. Bioact Mater 2024;31:119–35. 37637083 10.1016/j.bioactmat.2023.08.010 PMC10448243

[192] Eghosasere E , Osasumwen E, Emmanuella O. 3D bioprinting in tissue engineering: advancements, challenges, and pathways to clinical translation. JSM Regen Med Bio Eng 2025;7:1023.

[193] Zhang M , Zhang C, Li Z, Fu X, Huang S. Advances in 3D skin bioprinting for wound healing and disease modeling. Regen Biomater 2023;10:rbac105. 36683757 10.1093/rb/rbac105 PMC9845530

[194] Kammona O , Tsanaktsidou E, Kiparissides C. Recent developments in 3D-(bio)printed hydrogels as wound dressings. Gels 2024;10:147. 38391477 10.3390/gels10020147 PMC10887944

[195] Correa S , Grosskopf AK, Lopez Hernandez H, Chan D, Yu AC, Stapleton LM, Appel EA. Translational applications of hydrogels. Chem Rev 2021;121:11385–457. 33938724 10.1021/acs.chemrev.0c01177 PMC8461619

[196] Ge M , Guo Z, Ruan Z, Ma Y, Zhang Z, Dong H, Shi T, Hu T, Lu L, Chen Y, Lin H, Tan C. Chemical substrate-enabled piezoelectric metabolic reprogramming therapy unlocks the vicious triad of diabetic wounds. Cell Biomater 2025;1:100276.

[197] Chelu M , Calderon Moreno JM, Musuc AM, Popa M. Natural regenerative hydrogels for wound healing. Gels 2024;10:547. 39330149 10.3390/gels10090547 PMC11431064

[198] Zhu D , Hu Y, Kong X, Luo Y, Zhang Y, Wu Y, Tan J, Chen J, Xu T, Zhu L. Enhanced burn wound healing by controlled-release 3D ADMSC-derived exosome-loaded hyaluronan hydrogel. Regen Biomater 2024;11:rbae035. 38628545 10.1093/rb/rbae035 PMC11018541

[199] Aliakbar Ahovan Z , Esmaeili Z, Eftekhari BS, Khosravimelal S, Alehosseini M, Orive G, Dolatshahi-Pirouz A, Pal Singh Chauhan N, Janmey PA, Hashemi A, Kundu SC, Gholipourmalekabadi M. Antibacterial smart hydrogels: new hope for infectious wound management. Mater Today Bio 2022;17:100499. 10.1016/j.mtbio.2022.100499 PMC9709163 36466959

[200] Duong TKN , Truong TT, Phan TNL, Nguyen TX, Doan VHM, Vo TT, Choi J, Pal U, Dhar P, Lee B, Oh J, Mondal S. Hydrogel-based smart materials for wound healing and sensing. Aggregate 2025;6:e70047.

[201] Ma X , Zhu X, Lv S, Yang C, Wang Z, Liao M, Zhou B, Zhang Y, Sun S, Chen P, Liu Z, Chen H. 3D bioprinting of prefabricated artificial skin with multicomponent hydrogel for skin and hair follicle regeneration. Theranostics 2025;15:2933–50. 40083946 10.7150/thno.104854 PMC11898285

[202] Zhou K , Sun Y, Yang J, Mao H, Gu Z. Hydrogels for 3D embedded bioprinting: a focused review on bioinks and support baths. J Mater Chem B 2022;10:1897–907. 35212327 10.1039/d1tb02554f

[203] El-Husseiny HM , Mady EA, Hamabe L, Abugomaa A, Shimada K, Yoshida T, Tanaka T, Yokoi A, Elbadawy M, Tanaka R. Smart/stimuli-responsive hydrogels: cutting-edge platforms for tissue engineering and other biomedical applications. Mater Today Bio 2022;13:100186. 10.1016/j.mtbio.2021.100186 PMC8669385 34917924

[204] Ma H , Qiu C, Bao J, Jiang Y, Wang H, Zhang W, Zhao Q, Zhang Z, Tao H, Lu X, Zhang N, Zhu N. NIR-induced power-effective smart bandage for wound infection monitoring and accelerated healing. Nano Lett 2025;25:8203–10. 40354180 10.1021/acs.nanolett.5c01255

[205] Niu Y , Zhao Z, Yang L, Lv D, Sun R, Zhang T, Li Y, Bao Q, Zhang M, Wang L, Yan W, Han F, Yan B. Towards intelligent wound care: hydrogel-based wearable monitoring and therapeutic platforms. Polymers (Basel) 2025;17:1881. 40647892 10.3390/polym17131881 PMC12252240

